# Tumor-derived MG53 aggravates gastrointestinal cancer cachexia related muscle atrophy by inhibiting the TLR4/PI3K/AKT/FOXO3a signaling pathway

**DOI:** 10.3389/fonc.2026.1919475

**Published:** 2026-09-07

**Authors:** Yong-Fei Wang, Yuan-Yuan Xin, Si-Yuan Wang, Zi-Yi An, Dong-Yang Qi, Wen- Li, Wei-Lin Jin, Wen-Zhen Yuan

**Affiliations:** 1 Institute of Cancer Neuroscience, Medical Frontier Innovation Research Center, The First Hospital of Lanzhou University, The First Clinical Medical College of Lanzhou University, Lanzhou, China; 2 The First Clinical Medical College of Lanzhou University, Lanzhou, China; 3 Department of Surgical Oncology, The First Hospital of Lanzhou University, Lanzhou, China

**Keywords:** cancer cachexia, cancer cachexia factors, E3 ubiquitin ligases, inter-organ communication, MG53, muscle atrophy, PI3K/AKT/FOXO3a signaling pathway, skeletal muscle index

## Abstract

**Background:**

Cancer cachexia is a multisystem syndrome characterized by progressive skeletal muscle atrophy, anorexia, and weight loss, imposing a substantial global health burden. Gastrointestinal cancer cachexia is highly prevalent, yet its pathogenesis remains poorly understood, and there is currently a lack of effective diagnostic methods and therapeutic strategies specifically targeting muscle atrophy. Identifying diagnostic and therapeutic targets for gastrointestinal cancer cachexia-related muscle atrophy represents a critical challenge.

**Methods:**

This study investigated the role of MG53 in gastrointestinal cancer cachexia. Serum MG53 levels were analyzed in patients, and experimental models examined tumor-derived MG53 effects on cancer cachexia-related muscle atrophy.

**Results:**

Elevated serum MG53 levels were found in gastrointestinal cancer cachexia patients. Tumor-derived MG53 aggravates cancer cachexia-related muscle atrophy by inhibiting the TLR4/PI3K/AKT/FOXO3a signaling pathway in muscle.

**Conclusions:**

MG53 is identified as a novel cancer cachexia factor driving gastrointestinal cancer cachexia-related muscle atrophy by inhibiting the TLR4/PI3K/AKT/FOXO3a signaling pathway in muscle. MG53 correlates with muscle atrophy, supporting its potential as a diagnostic biomarker and therapeutic target of gastrointestinal cancer cachexia.

## Graphical Abstract

**Figure:**
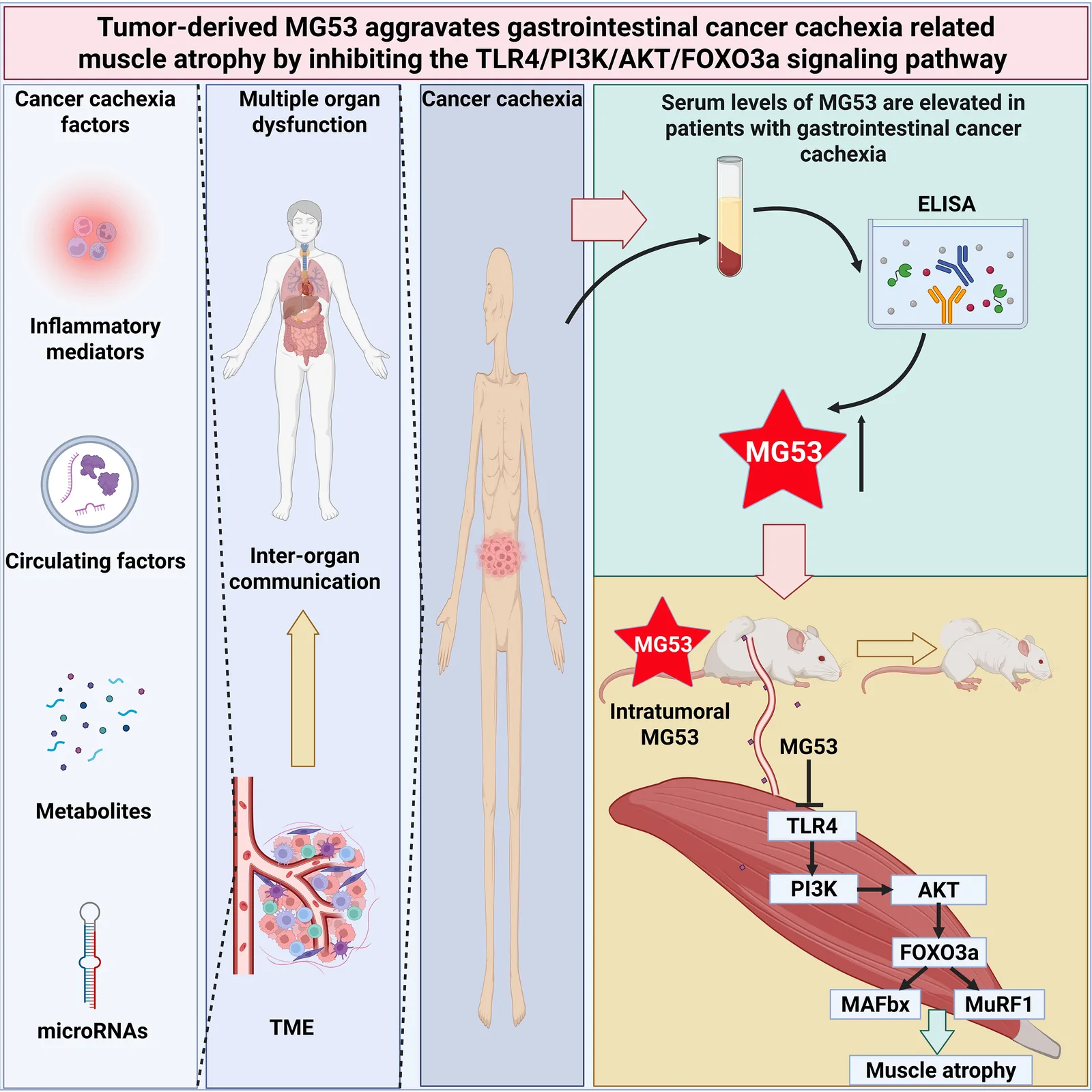
This graphical abstract illustrates the mechanism by which MG53 aggravates gastrointestinal cancer cachexia-related muscle atrophy. The development of cancer cachexia involves multiple organ dysfunction, driven by inflammatory mediators, circulating factors, metabolites, and microRNAs. Our study found that serum MG53 levels are elevated in patients with gastrointestinal cancer cachexia. Tumor-derived MG53 aggravates cancer cachexia-related muscle atrophy by inhibiting the TLR4/PI3K/AKT/FOXO3a signaling pathway in muscle, thereby upregulating the expression of MAFbx and MuRF1 proteins, ultimately aggravates cancer cachexia-related muscle atrophy.

## 1 Introduction

The term cachexia originates from the Greek words kakos (bad) and hexis (condition). Cachexia is a syndrome characterized by severe wasting, often associated with cancer, and marked by muscle loss with or without lipolysis (1, 2). Cancer cachexia arises as a consequence of most malignant tumors, exhibiting a high incidence and mortality rate. The prevalence of cachexia in common malignancies ranges from 20% to 90%, and approximately 22% of cancer patients ultimately succumb to cachexia. Among these, gastrointestinal cancer cachexia is particularly prevalent, with incidence rates reported as high as 80–90% in gastric, pancreatic, and colorectal cancers (3, 4). This condition imposes a significant burden on global healthcare systems and profoundly negatively impacts patients’ treatment response, quality of life, and long-term survival (5). Moreover, the pathogenesis of cancer cachexia remains unclear, and there are currently only limited symptomatic therapies such as appetite improvement, which fail to meet clinical needs (4, 6). Therefore, an updated understanding of the driving mechanisms of cachexia is needed to provide targeted therapies for patients.

Cancer cachexia is a multi-organ syndrome, and its development is closely associated with alterations in inter-organ signaling communication (7), mediated by cachexia-inducing factors such as inflammatory mediators, circulating factors, metabolites, and microRNAs (8–10). Irreversible muscle atrophy represents the most characteristic clinical manifestation, leading to a key focus on the interplay between tumors and muscle as a potential diagnostic and therapeutic avenue (11).

The pathogenesis of this muscle atrophy is highly complex, resulting from reduced protein synthesis, enhanced proteolysis, or both, with the ubiquitin-proteasome system playing a critical role in controlling muscle mass (12, 13). Protein degradation is associated with increased levels of E3 ubiquitin ligases, such as MuRF1 and MAFbx, which are highly expressed during atrophy (14). Although research on targets for cancer cachexia-related muscle atrophy is currently very active, interventions directed at these known targets have shown only limited efficacy, underscoring the pressing need for the identification of new and specific targets for cancer cachexia-related muscle atrophy.

MG53 is a member of the TRIM family of proteins, also known as tripartite motif-containing protein 72 (TRIM72) (hereafter referred to as MG53). MG53 is an E3 ubiquitin ligase, mainly expressed in cardiac and skeletal muscles (15). MG53 exhibits complex biological functions due to its dual roles in plasma membrane repair and E3 ubiquitin ligase activity. Current studies indicate that MG53 exerts protective effects in multiple organ systems and demonstrates anti-tumor properties in certain cancers (16). However, alongside these beneficial roles, MG53 has also been implicated in adverse effects such as negative inotropy, insulin resistance, and metabolic syndrome (17). This article focuses on the negative inotropic effect of MG53. Available evidence suggests that MG53 expression is regulated by the IGF/IRS-1/PI3K/AKT/MyoD signaling pathway, and once expressed, MG53 interacts with IRS-1 to negatively regulate muscle formation (17–20). Therefore, we speculate that MG53 may lead to muscle atrophy in cancer cachexia.

Our study confirmed that MG53 is a potential biomarker for gastrointestinal cancer cachexia, with elevated serum levels correlating with muscle atrophy. Mechanistically, tumor-derived MG53 aggravates muscle atrophy by inhibiting the TLR4/PI3K/AKT/FOXO3a signaling pathway, thereby promoting proteolysis and suppressing protein synthesis. These findings highlight MG53 not only as a diagnostic biomarker but also as a promising therapeutic target for counteracting cachexia-related muscle atrophy in gastrointestinal cancer patients.

## 2 Materials and methods

### 2.1 Patients and serum sample collection

Serum samples were collected from patients with gastrointestinal tumors admitted to the oncology department, radiotherapy department, and general surgery department of the first hospital of Lanzhou university between September 2022 and December 2023. Serum was obtained by centrifuging whole blood at 3000 revolutions per minute for 15 minutes. After centrifugation, the serum samples were sequentially numbered according to the order of collection and immediately stored at –80 °C for subsequent analysis.

Inclusion criteria: The diagnostic criteria for cancer cachexia were based on the clinical diagnosis and treatment guidelines for cancer cachexia (2020 Edition): 1. Weight loss >5% within six months without dieting; 2. BMI <20 kg/m^2^ (for Caucasians), BMI <18.5 kg/m^2^ (for Chinese), and any degree of weight loss >2% within six months; 3. Appendicular skeletal muscle index (ASMI) meeting the criteria for sarcopenia (male <7.26 kg/m^2^, female <5.45 kg/m^2^) and any degree of weight loss >2% (according to the European Palliative Care Research Association standards); 4. Reduced food intake/systemic inflammation. Diagnosis can be made if one of criteria 1, 2, or 3 is met in addition to criterion 4. This study was approved by the institutional review board of the first hospital of Lanzhou university, approval number: LDYYLL2024-223. The research followed guidelines of the Declaration of Helsinki and Tokyo for humans, and was approved by the institutional human experimentation committee or equivalent, and that informed consent was obtained.

Exclusion criteria: Current research has found that serum MG53 levels are elevated in patients with diabetes and myocardial infarction (21, 22). To avoid the impact of these diseases on the results, we excluded patients with cancer cachexia who also had diabetes or myocardial infarction.

### 2.2 Assessment of skeletal muscle mass

A single-slice CT image at the level of the third lumbar vertebra (L3) was selected for body composition analysis. The image was analyzed by a trained researcher using Slice-Omatic 5.0 software (TomoVision, Montreal, Canada) to calculate the cross-sectional area of specific tissue types. Additionally, tissue-specific Hounsfield unit (HU) thresholds of -29 to 150 were applied to assess the L3 skeletal muscle area (23). The total muscle area was normalized to the patient’s height and reported as the skeletal muscle index (SMI; cm^2^/m^2^). The L3 skeletal muscle index (SMI) was calculated using the formula: total L3 skeletal muscle area (cm^2^)/height squared (m^2^) (24, 25). Previous studies classified patients with sarcopenia based on established thresholds: male <52.4 cm^2^/m^2^, female <38.5 cm^2^/m^2^ (26–29). However, these criteria were established for Western populations and may not be applicable to Asian populations. Therefore, we adopted prognosis-based SMI cut-off values from previous research, defining reduced muscle mass as male <40.8 cm^2^/m^2^ and female <34.9 cm^2^/m^2^ (30).

### 2.3 Protein expression and purification

The expression and purification of the rhMG53 protein were performed according to a previously described method (31). The plasmid encoding human MG53 [pGEX-4T-1-TRM72 (MG53) C14A] was constructed (Jingmai, China), and the fusion protein rhGST-MG53 (hereinafter referred to as rhMG53) was purified using glutathione agarose resin (Yeasen, China) via affinity chromatography. The size and purity of the recombinant protein were analyzed by sodium dodecyl sulfate–polyacrylamide gel electrophoresis (SDS-PAGE), and aliquots of the protein were stored at −80 °C.

### 2.4 Cell culture and treatment

CT26 cells and C2C12 myoblasts were provided by the laboratories of Dong-ping Wei (Nanjing Medical University) and Dong-hai Lin (Xiamen University), respectively. HEK-293FT, HCT116, and MC38 cells were from Shanghai Jiao Tong University. CT26, C2C12, HEK-293FT, and MC38 cells were cultured in high-glucose DMEM (Corning, USA) with 10% FBS (XY Cell) and 1% penicillin/streptomycin (Yeasen, China). HCT116 cells were cultured in RPMI-1640 medium (Corning, USA) with the same supplements. All cells were maintained at 37 °C in a 5% CO_2_ incubator.

### 2.5 Cancer cachexia models *in vitro*

The cancer cachexia *in vitro* model was established as previously described (32). CT26 or MC38 cells (5×10^5^) were seeded into 100 mm dishes. At 90% confluence, medium was replaced with serum-free medium. After 24 h, conditioned medium (CM) was collected, centrifuged (1200 rpm, 10 min), and filtered (0.2 μm). The obtained CMs—CM(CT26-shNC-MG53), CM(CT26-shMG53), CM(CT26-VECTOR-MG53), CM(CT26-OE-MG53), CM(MC38-VECTOR-MG53), and CM(MC38-OE-MG53)—were aliquoted and stored at −80 °C. For atrophy induction, CM was mixed 1:1 with differentiation medium containing horse serum and added to C2C12 myotubes on day 3 of differentiation.

### 2.6 Differentiation of C2C12 myoblasts

The differentiation of C2C12 myoblasts was performed referring to the previous experimental method (33). C2C12 myoblasts were seeded in 6-well plates (2×10^5^ cells/well) and 24-well plates (1×10^5^ cells/well). Then, when the C2C12 myoblasts reached 80% confluence, the medium was replaced with differentiation medium containing 2% horse serum. After 6 days, the C2C12 myoblasts differentiated into myotubes, which were used for WB and immunofluorescence staining.

### 2.7 WB

WB analysis was performed according to a previously described method (34). Cells were lysed in high-potassium lysis buffer (34), and the protein concentration in the samples was measured using a protein concentration assay kit (Solarbio, China). Protein samples (30 μg) were loaded and separated by 8%, 10%, or 12% polyacrylamide gel electrophoresis based on the molecular weight of the target protein, and then transferred to a PVDF membrane (Amersham Hybond P 0.45, Germany). After blocking with 5% skim milk for 2 hours, the membranes were incubated with primary antibodies at 4 °C overnight. The next day, HRP-conjugated secondary antibodies (1:5,000; Abmart) were added and incubated at room temperature for 1 hour. Detection was performed using chemiluminescence (Amersham Imager 680, USA), and grayscale values were calculated and analyzed using Image J software. The antibodies used are listed in **Table 1**.

**Table 1:** Antibodies.

Name	Company	Catalog number
Myogenin	ABclonal	A17427
MyHC(myosin heavy chain)(WB)	ABclonal	A4963
MuRF1(mLlselering-finger)	ABclonal	A3101
MAFbx (muscle atrophy F-box)	ABclonal	A3193
MG53 (TRIM72)	ABclonal	A16751
MyHC(myosin heavy chain)(IF)	R&D Systems	MAB4470
AKT	Abmart	P31749
P-AKT	Abmart	P31749
PI3K	Abmart	P27986
P-PI3K	Abmart	P27986
FOXO3a	Abmart	O43524
P-FOXO3a	Abmart	O43524
GAPDH	Abmart	M20006
β-Actin	Abmart	T40104
Goat Anti-Rabbit IgG-HRP	Abmart	AB2713951
Goat Anti-Mouse IgG-HRP	Abmart	AB2713950
Alexa Fluor 594	Abmart	M213608S
Alexa Fluor 488	Abmart	M213208S

### 2.8 RNA extraction and qRT-PCR

Total RNA was fractioned using TRIZOL (Ambion) according to the manufacturer’s instructions. Total RNA was fractioned from CT26-shNC-MG53 and CT26-shMG53 cells, C2C12-shNC-MG53 and C2C12-shMG53 myoblasts, CT26-VECTOR-MG53 and CT26-OE-MG53-cells, and MC38-VECTOR-MG53 and MC38-OE-MG53 cells using TRIzol reagent. The RNA was then reverse-transcribed into cDNA using the Hifair^®^ III 1st Strand cDNA Synthesis SuperMix kit (Yeasen, China). PCR amplification was performed using the Hieff^®^ qPCR SYBR Green Master Mix (No Rox) kit (Yeasen, China). The primer sequences were as follows: MG53, 5’-ACTGAGCATCTACTGCGAGC-3’ and 5’-ACGATGACCACGGTGAGAAC-3’; GAPDH, 5’-ACCACAGTCCATGCCATCAC-3’ and 5’-TCCACCACCCTGTTGCTGTA-3’.

### 2.9 Immunofluorescence staining

Immunofluorescence staining was performed as previously described (35). C2C12 cells (2×10^4^/well) were seeded on coated glass slides. At 80% confluence, medium was replaced with differentiation medium containing 2% horse serum (Solarbio, China). On day 3 of differentiation, 0.2 μM rhMG53 or various conditioned media (CM) were added for 72 h. Cells were washed with PBS, fixed with 4% paraformaldehyde (15 min), then ice-cold methanol (15 min), blocked with Ab buffer (45 min, RT), and incubated with anti-MyHC antibody (MF20, 25 μg/ml, R&D Systems) at 4 °C overnight. After washing, cells were incubated with Alexa Fluor 594 or 488 secondary antibodies (1:200, Abmart) for 2 h at RT, washed, stained with DAPI (5 μg/ml, Yeasen) for 5 min in the dark, washed, and mounted. Images were acquired at 200× magnification.

### 2.10 Lentiviral packaging and infection

MG53-specific shRNA sequences were designed and used to construct plasmids. HEK-293FT cells were thawed and cultured. Using the Lentiviral Packaging Kit (Yeasen Biotechnology Co., Ltd., Shanghai, China) the plasmids were transfected into 293FT cells, and the viral supernatant was collected. When CT26 and C2C12 cells reached 50% confluence, they were infected with the lentivirus. On the third day post-infection, 2 µg/mL puromycin was added for selection over 8 days. The cells were then frozen for future use. The sequence of the MG53-specific shRNA is as follows: mMG53-shRNA-1#: GAG CTG TCA AGC CTG AAC TCT.

### 2.11 Animal

Male BALB/c and C57BL/6 mice (6–8 weeks old, from Lanzhou Veterinary Research Institute, Chinese Academy of Agricultural Sciences). Mice were deeply anesthetized with an intraperitoneal injection of sodium pentobarbital (50 mg/kg). Once the pedal reflex was absent, euthanasia was performed by cervical dislocation. Death was confirmed by cessation of respiration and pupil dilation. The housing and experimental protocols of this study comply with relevant Chinese regulations and the Guide for the Care and Use of Laboratory Animals issued by the National Institutes of Health (NIH), and have been approved by the Animal Care and Use Committee of the First Hospital of Lanzhou University (Approval No.: LDYYLL2026-608).

### 2.12 Cancer cachexia model *in vivo*

Mice were housed under constant temperature (22 ± 2 °C) with a 12-hour light/dark cycle (five mice per cage) and free access to food and distilled water. After one week of acclimatization, they were randomly divided into three groups: a non-tumor healthy group (Healthy, 10 mice), a CT26 tumor-bearing group (CT26-shNC-MG53, 10 mice), and a CT26 tumor-bearing group with MG53 knockdown (CT26-shMG53, 10 mice). CT26 cells (1.0×10^6^ per mouse) were inoculated subcutaneously. Daily records of food intake, body weight, and tumor growth were maintained. At the end of the experiment, serum, tumor tissues, gastrocnemius, and tibialis anterior muscles were collected. The experiment for CT26 tumor-bearing mice overexpressing MG53 followed the same procedure. For MC38 tumor-bearing mice overexpressing MG53, C57BL/6 mice were used, with grouping and experimental methods identical to those described above.

### 2.13 Muscle tissue collection

At the end of the experiment, the mice were weighed and fasted for 24 hours. After euthanasia, bilateral hindlimb gastrocnemius muscles were harvested and weighed, then stored at -80 °C for subsequent histopathological analysis and WB.

### 2.14 Histological observation

Gastrocnemius muscle tissues were fixed in 4% paraformaldehyde for 24 hours, dehydrated, embedded in paraffin, and sectioned into 5 µm-thick slices. The sections were then subjected to hematoxylin and eosin (HE) staining. HE-stained sections were scanned using a fully automated slide scanner.

### 2.15 Enzyme-linked immunosorbent assay

MG53 levels were measured using Human TRIM72 ELISA Kit (Baililai, China) for human serum and Mouse TRIM72 ELISA Kit (Baililai, China) for mouse serum and cell culture supernatants, following the manufacturer’s instructions.

### 2.16 Statistical analysis

Results are expressed as the mean ± standard deviation (SD). Differences between groups were analyzed for statistical significance using one-way analysis of variance (ANOVA), followed by *post-hoc* multiple comparison tests (Tukey’s HSD test) conducted with Prism version 8.0 (GraphPad, CA, USA). For all analyses, *p < 0.05, **p < 0.01, ***p < 0.001, and ****p < 0.0001 were considered statistically significant.

## 3 Results

### 3.1 Serum levels of MG53 are higher in patients with gastrointestinal cancer cachexia compared to non-cachectic cancer patients

#### 3.1.1 Baseline characteristics

A total of 937 serum samples from patients with gastrointestinal cancer were collected. According to the screening and exclusion criteria for cancer cachexia, 172 serum samples from cancer cachexia patients were obtained, and 78 serum samples from non-cachectic cancer patients were randomly matched. The demographic and laboratory characteristics of the patients are shown in **Table 2**. Compared with non-cachectic cancer patients, cancer cachexia patients had higher C-reactive protein (CRP) levels (p < 0.001), which is consistent with previous research findings (36). However, white blood cell (WBC) count (p = 0.18) and neutrophil-to-lymphocyte ratio (NLR) (p = 0.41) did not differ significantly from those of non-cachectic patients. In contrast, previous studies have reported decreased serum levels of prealbumin (PAB) and albumin (ALB) in patients with cancer cachexia (37). Statistical analysis of our results revealed that serum PAB levels were lower in gastrointestinal cancer cachexia patients compared to non-cachectic patients (p = 0.0449), but there was no significant difference in ALB levels (p = 0.0521). Additionally, no significant differences were observed between the two groups in terms of gender, body mass index, disease type, or tumor stage.

**Table 2:** Baseline clinical characteristics of patients with gastrointestinal tumors.

	Total cancer (n=250)	No cachexia (n=78)	Cachexia (n=172)	P-value
Age (year)		57.60 ± 10.61	61.68 ± 10.66	P=0.12
Sex				
Male	172 (68.80%)	55 (70.51%)	117 (68.02%)	
Female	78 (31.20%)	23 (29.49%)	55 (31.98%)	
BMI (kg/m^2^)	20.99 ± 3.45	21.39 ± 2.88	20.81 ± 3.68	P=0.22
Primary malignancy				
Esophagus	11 (4.40%)	1 (1.28%)	10 (5.81%)	
Stomach	145 (58.00%)	42 (53.85%)	103 (59.88)	
Duodenum	2 (0.80%)	1 (1.28%)	1 (0.58%)	
Colorectal	74 (29.60%)	26 (33.33%)	48 (27.91%)	
Hepatocellular	10 (4.00%)	5 (6.41%)	5 (2.91%)	
Biliary	2 (0.80%)	2 (2.56%)	0 (0%)	
Pancreatic	2.40 (2.6%)	1 (1.28%)	5 (2.91%)	
Stage				P=0.42
I	5 (2.00%)	2 (2.56%)	3 (1.74%)	
II	26 (10.40%)	7 (8.97%)	19 (11.05%)	
III	44 (17.60%)	22 (28.21%)	22 (12.79%)	
IV	47 (18.80%)	15 (19.23%)	32 (18.60%)	
Unknown	128 (51.20%)	32 (41.03%)	96 (55.81%)	
SMI (cm^2^/m^2^)	41.11 ± 10.39	44.53 ± 10.42	39.44 ± 9.99	P<0.001
Sarcopenia	105 (42.00%)	39 (50.00%)	66 (42.86%)	
PAB (mg/L)	180.10 ± 61.41	197.80 ± 61.98	172.00 ± 59.60	P=0.0021
ALB (g/L)	41.53 ± 0.30	42.37 ± 5.22	41.06 ± 4.77	P=0.0521
Hypoproteinemia	25 (10.04%)	8 (10.39%)	17 (9.88%)	
WBC (10^9/L)	5.56 ± 3.04	5.58 ± 2.64	5.55 ± 3.21	p=0.9473
CRP (mg/L)	11.64 ± 20.62	4.58 ± 7.62	14.87 ± 23.74	P=0.0340
N (10^9/L)	3.70 ± 2.13	3.70 ± 2.44	3.70 ± 1.99	P=0.9979
L (10^9/L)	1.26 ± 0.54	1.35 ± 0.61	1.22 ± 0.51	P=0.06
NLR (N/L)	3.59 ± 3.33	3.26 ± 0.36	3.72 ± 0.24	P=0.38

WBC, white blood cell count; CRP, C-reactive protein; N, neutrophil; L lymphocyte ratio; NLR, neutrophil-to-lymphocyte ratio; PAB, prealbumin; ALB, albumin.

#### 3.1.2 Serum levels of IL-6 and TNF-α are higher in patients with gastrointestinal cancer cachexia than in non-cachectic cancer patients

**Figure 1:**
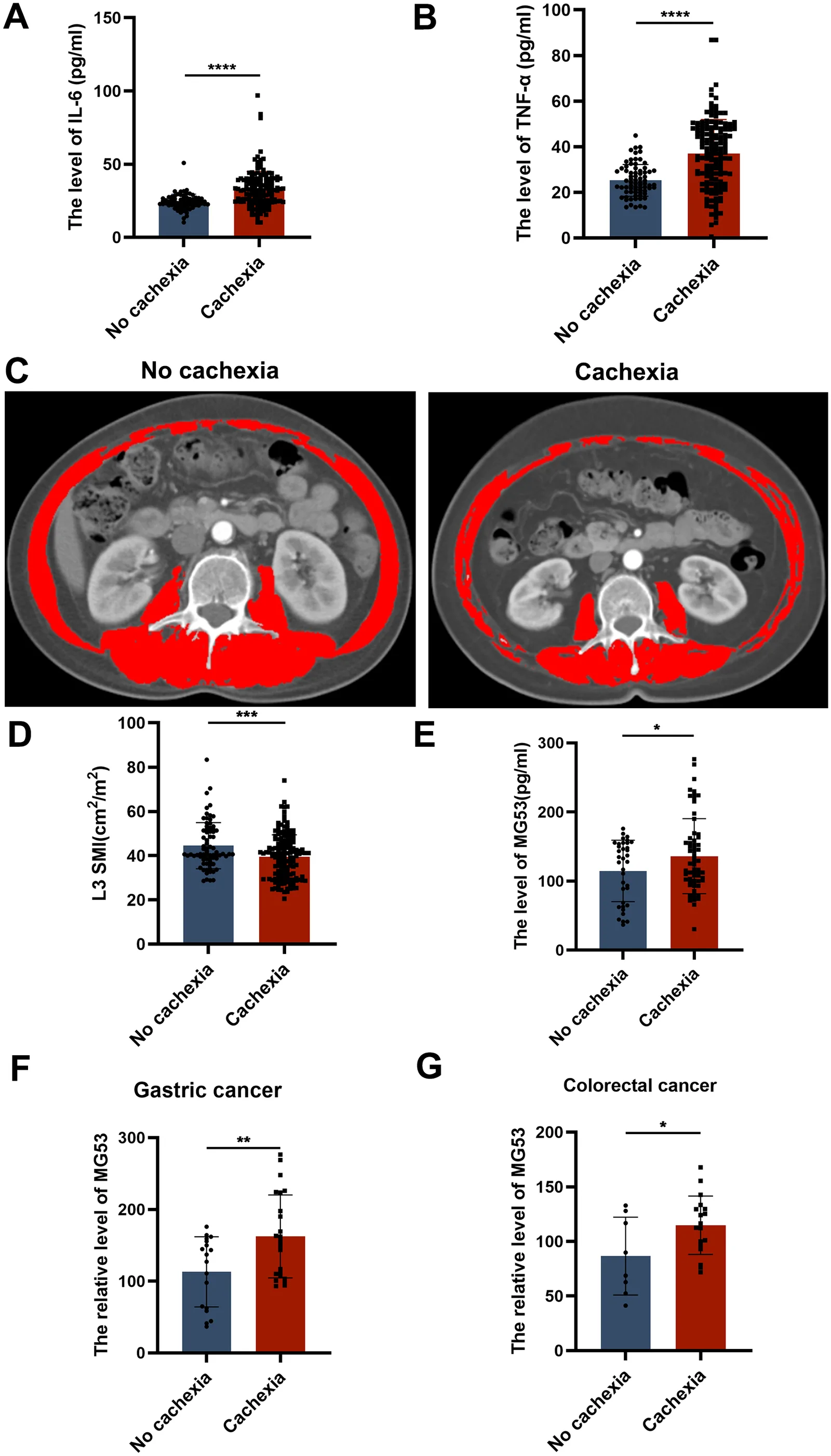
Detection of serum MG53 levels in patients with gastrointestinal cancer cachexia and without cachexia. **(A)** Detection of serum IL-6 levels in patients with gastrointestinal cancer with and without cachexia (No cachexia 78, cachexia 172; p <0.0001); **(B)** Detection of serum TNF-α levels in patients with gastrointestinal cancer with and without cachexia (No cachexia 78, cachexia 172; p <0.0001); **(C)** Schematic diagram of L3 skeletal muscle area analysis in patients with gastrointestinal cancer with and without cachexia using Slice-Omatic 5.0 software. The skeletal muscle is shown in red (tissue-specific Hounsfield unit (HU) threshold: -29 to 150); **(D)** Statistical analysis of L3 skeletal muscle index in patients with gastrointestinal cancer with and without cachexia (No cachexia 75, cachexia 154; p <0.001). **(E)** Serum MG53 levels in patients with gastrointestinal cancer-associated cachexia versus non-cachectic patients (No cachexia 36, cachexia 64; p <0.05). **(E)** Serum MG53 levels in gastric cancer patients with cachexia versus non-cachectic patients (No cachexia 18, cachexia 24; p <0.01). **(F)** Serum MG53 levels in colorectal cancer patients with cachexia versus non-cachectic patients (No cachexia 8, cachexia 18; p <0.05). Results are expressed as the mean ± standard deviation (SD). Differences between groups were analyzed for statistical significance using one-way analysis of variance (ANOVA), followed by *post-hoc* multiple comparison tests (Tukey’s HSD test) conducted with Prism version 8.0 (GraphPad, CA, USA). For all analyses, *p < 0.05, **p < 0.01, ***p < 0.001, and ****p < 0.0001 were considered statistically significant.

Previous studies have shown that serum levels of IL-6 and TNF-α are higher in cancer cachexia patients than in non-cachectic cancer patients (36). To further validate the accuracy of our grouping, human IL-6 and TNF-α ELISA kits were used to measure the serum levels of IL-6 and TNF-α in 172 patients with gastrointestinal cancer cachexia and 78 patients with gastrointestinal cancer without cachexia. It was found that serum IL-6 levels were higher in patients with gastrointestinal cancer cachexia than in non-cachectic patients (**Figure 1A**), and serum TNF-α levels were also higher in patients with gastrointestinal cancer cachexia than in non-cachectic patients (**Figure 1B**).

#### 3.1.3 Patients with gastrointestinal cancer cachexia exhibit a lower L3 skeletal muscle index but higher serum MG53 levels compared to non-cachectic patients

Recent studies have shown that cancer cachexia patients exhibit a reduced L3 skeletal muscle index (38). To further validate the accuracy of our grouping, we compared the L3 skeletal muscle index between 172 patients with gastrointestinal cancer cachexia and 78 non-cachectic patients, and found it to be significantly lower in the cachectic group (**Figures 1C, D**). Subsequently, based on previous studies (diagnostic criteria for sarcopenia: men <52.4 cm^2^/m^2^, women <38.5 cm^2^/m^2^), we further refined the grouping by selecting cachectic patients who met the criteria for sarcopenia and non-cachectic patients who did not meet the criteria, and detected serum MG53 levels. It was found that serum MG53 levels were higher in patients with gastrointestinal cancer cachexia than in non-cachectic patients (**Figure 1E**). Furthermore, we separately analyzed patients with gastric cancer and colorectal cancer, and found that serum MG53 levels were significantly elevated in those with cachexia compared to non-cachexia patients in both groups (**Figures 1F, G**).

In this section, we collected serum samples from patients with gastrointestinal cancer. After excluding those with diabetes or myocardial infarction, the remaining samples were divided into two groups based on body weight: cancer cachexia and non-cachexia. One study noted that assessing either weight loss grading or SMI alone is insufficient to fully define cachexia; thus, both indicators are considered diagnostic criteria (39). The use of SMI helps overcome multiple limitations associated with clinical weight recording, such as inconsistent timeframes, insufficient precision, recall bias, and missing data (39). Therefore, after initially screening patients into cachexia and non-cachexia groups based on body weight, we further refined the selection using two dimensions: inflammatory markers and skeletal muscle index. Ultimately, our study revealed that serum MG53 levels were higher in patients with gastrointestinal cancer cachexia than in non-cachectic patients.

### 3.2 Exogenous MG53 can induce muscle atrophy

**Figure 2:**
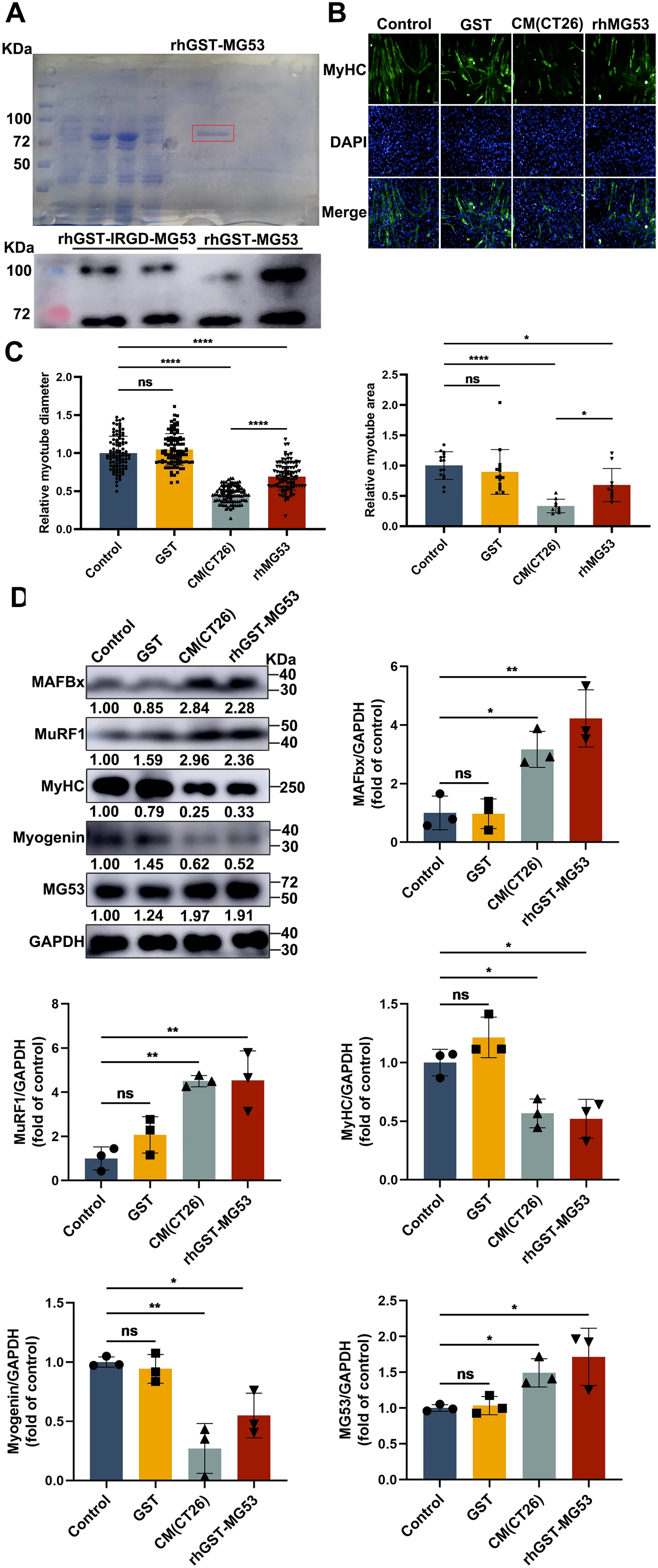
Changes in protein expression levels and MyHC immunofluorescence staining in C2C12 myotubes treated with rhMG53 protein. **(A)** Coomassie blue staining and WB validation of purified rhMG53 protein; **(B)** MyHC immunofluorescence staining of C2C12 myotubes. Scale bar, 100 μm; **(C)** Protein expression levels of MG53, Myogenin, MyHC, MuRF1, and MAFbx in myotubes detected by WB. Relative quantification of muscle atrophy- and synthesis-related proteins was performed using Image J software; **(D)** Statistical analysis of myotube diameter and area from immunofluorescence staining measured by ImageJ software. Results are expressed as the mean ± standard deviation (SD). Differences between groups were analyzed for statistical significance using one-way analysis of variance (ANOVA), followed by *post-hoc* multiple comparison tests (Tukey’s HSD test) conducted with Prism version 8.0 (GraphPad, CA, USA). For all analyses, *p < 0.05, **p < 0.01, ***p < 0.001, and ****p < 0.0001 were considered statistically significant.

In Section 3.1, we observed that gastrointestinal cancer patients with cachexia had significantly higher serum MG53 levels compared to non-cachectic patients. This finding prompted us to investigate whether MG53 induces muscle atrophy. To this end, we expressed and purified recombinant GST-tagged MG53 (rhGST-MG53), which was confirmed by Coomassie staining and Western blotting (**Figure 2A**). We then used a C2C12 *in vitro* myotube model and treated the differentiated myotubes with purified rhMG53 protein. Immunostaining for MyHC at differentiation day 6 showed that myotube diameter and area were significantly decreased (**Figures 2B, C**). WB analysis revealed increased MG53, MuRF1, and MAFbx, with decreased Myogenin and MyHC (**Figure 2D**). These results indicate that exogenous MG53 can induce atrophy *in vitro* myotubes.

**Figure 3:**
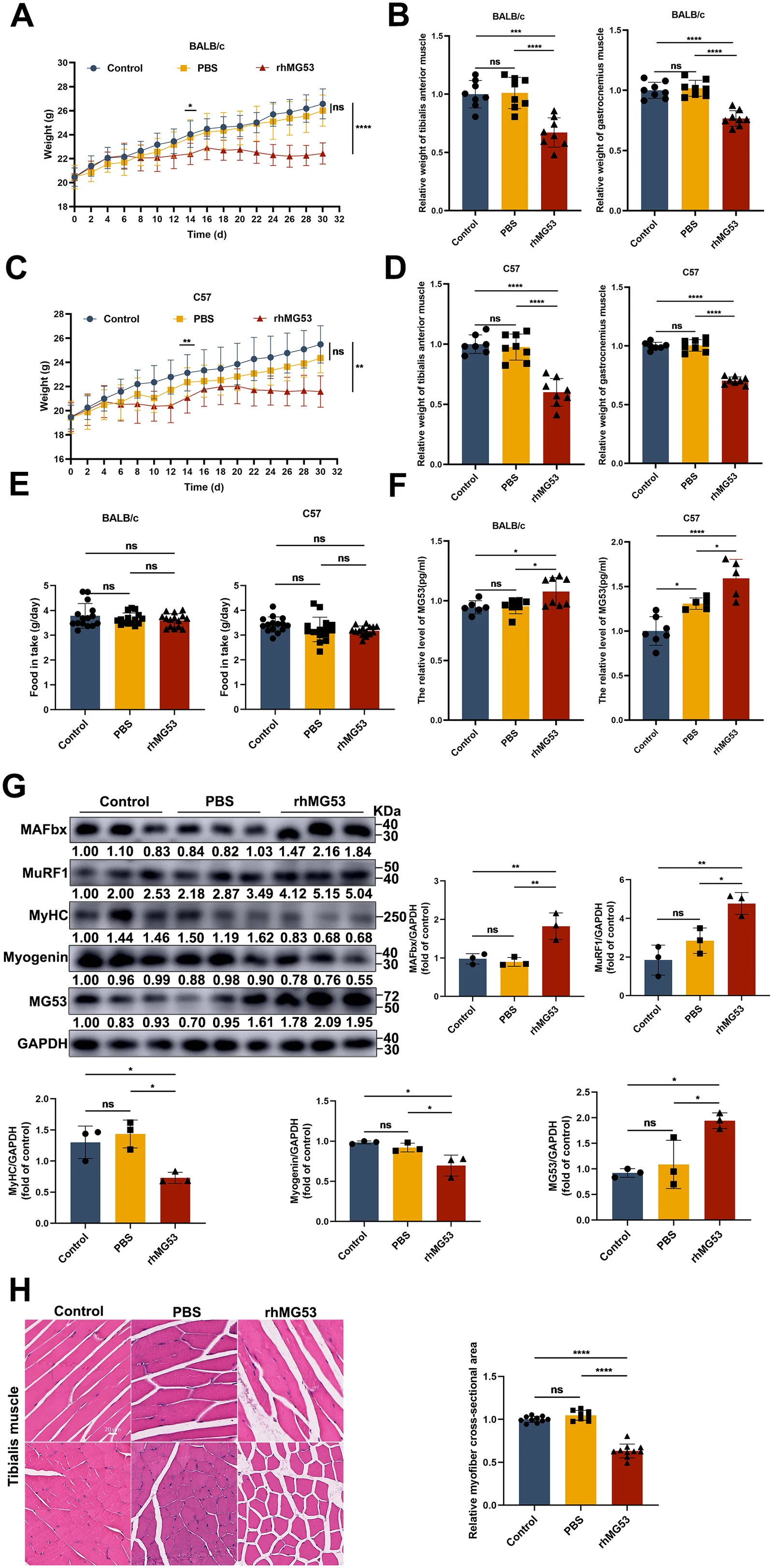
Intraperitoneal injection of rhMG53 protein induces muscle atrophy in mice. **(A)** Body weight growth curves of BALB/c mice in the rhMG53-treated, blank control, and negative control groups (n=10, p <0.0001); **(B)** Weights of the tibialis anterior and gastrocnemius muscles in BALB/c mice from the rhMG53-treated, blank control, and negative control groups (n=10, p <0.0001); **(C)** Body weight growth curves of C57BL/6 mice in the rhMG53-treated, blank control, and negative control groups (n=10, p <0.01); **(D)** Weights of the tibialis anterior and gastrocnemius muscles in C57BL/6 mice from the rhMG53-treated, blank control, and negative control groups (n=10, p <0.0001); **(E)** Changes in food intake of BALB/c and C57BL/6 mice in the rhMG53-treated, blank control, and negative control groups (n=10, ns); **(F)** Serum MG53 levels in BALB/c and C57BL/6 mice from the rhMG53-treated, blank control, and negative control groups (n=10, p <0.05; n=10, p <0.0001); **(G)** Protein expression levels of MG53, Myogenin, MyHC, MuRF1, and MAFbx in the tibialis anterior muscle of BALB/c mice detected by WB. Relative quantification of muscle atrophy- and synthesis-related proteins was performed using ImageJ software (n=3); **(H)** HE staining of the tibialis anterior muscle in BALB/c mice (n=3). Results are expressed as the mean ± standard deviation (SD). Differences between groups were analyzed for statistical significance using one-way analysis of variance (ANOVA), followed by *post-hoc* multiple comparison tests (Tukey’s HSD test) conducted with Prism version 8.0 (GraphPad, CA, USA). For all analyses, *p < 0.05, **p < 0.01, ***p < 0.001, and ****p < 0.0001 were considered statistically significant.

BALB/c and C57BL/6 mice received rhMG53 via intraperitoneal injection (1 mg/kg, every two days), while negative controls received equal volumes of PBS and blank controls received no treatment. Body weight and food intake were measured every two days for 30 days. In BALB/c mice, the rhMG53-treated group showed significantly decreased body weight from around day 14 compared to controls (**Figure 3A**), along with reduced tibialis anterior and gastrocnemius muscle weights (**Figure 3B**). Similar results were observed in C57BL/6 mice (**Figures 3C, D**). However, no changes in food intake were observed in either group of mice (**Figure 3E**). At the end of the experiment, serum and muscle tissues were collected from the mice. Serum MG53 levels were detected using a mouse MG53 ELISA kit, and it was found that MG53 levels in the serum of both BALB/c and C57BL/6 mice in the rhMG53-treated group were higher than those in the blank and negative control groups (**Figure 3F**). WB analysis of tibialis anterior muscle tissue (BALB/c) showed that MG53 protein expression increased in the rhMG53-treated group, while the expression of muscle synthesis-related proteins Myogenin and MyHC decreased, and the expression of muscle atrophy-related proteins MuRF1 and MAFbx increased (**Figure 3G**). The same results were also observed by WB analysis of gastrocnemius muscle tissue from BALB/c mice (**Supplementary Figure 1A**) as well as tibialis anterior muscle (**Supplementary Figure 1C**) and gastrocnemius muscle (**Supplementary Figure 1E**) tissue from C57BL/6 mice. Additionally, HE staining showed disordered arrangement and reduced fiber diameter in tibialis anterior of rhMG53-treated BALB/c mice (**Figure 3H**), and similar changes were seen in BALB/c gastrocnemius and C57BL/6 tibialis anterior and gastrocnemius (**Supplementary Figures 2A–C**). These findings indicate that exogenous MG53 protein, absorbed into the bloodstream and entering muscle tissue, induces muscle atrophy in mice.

### 3.3 Endogenous MG53 can cause atrophy in C2C12 myotubes

Having observed that exogenous MG53 induces C2C12 myotube atrophy *in vitro*, we next investigated the role of endogenous MG53. To this end, we generated MG53-knockdown C2C12 cell lines (C2C12-shNC-MG53 and C2C12-shMG53) and confirmed knockdown efficiency by Western blotting and qRT-PCR (**Supplementary Figure 3A**). Immunostaining for MyHC at differentiation day 6 showed that myotube diameter and area were significantly increased in the C2C12-shMG53 group compared to controls (**Supplementary Figures 3B, C**). Then, we conducted WB experiments on C2C12 myotubes at day 6 of differentiation, which revealed that the expression levels of MuRF1 and MAFbx decreased, while the expression levels of Myogenin and MyHC increased (**Supplementary Figure 3D**). Subsequently, we also constructed MG53-overexpressing C2C12 lines (C2C12-VECTOR-MG53 and C2C12-OE-MG53), confirmed by WB and qRT-PCR (**Supplementary Figure 3E**). Next, we performed immunofluorescence analysis on C2C12 myotubes at day 6 of differentiation to examine MyHC expression, and measured the myotube diameter and area using Image J software. The results showed that the myotube diameter and area in the C2C12-OE-MG53 group was significantly decreased compared to the C2C12-VECTOR-MG53 group (**Supplementary Figures 3F, G**), with increased MuRF1 and MAFbx and decreased Myogenin and MyHC by WB (**Supplementary Figure 3H**). These results indicate that endogenous MG53 also induces myotube atrophy, consistent with previous studies showing MG53 negatively regulates muscle synthesis (18).

### 3.4 Tumor-derived MG53 aggravates cancer cachexia-associated muscle atrophy

#### 3.4.1 Atrophy of C2C12 myotubes induced by supernatant from MG53-knockdown CT26 cells is alleviated

**Figure 4:**
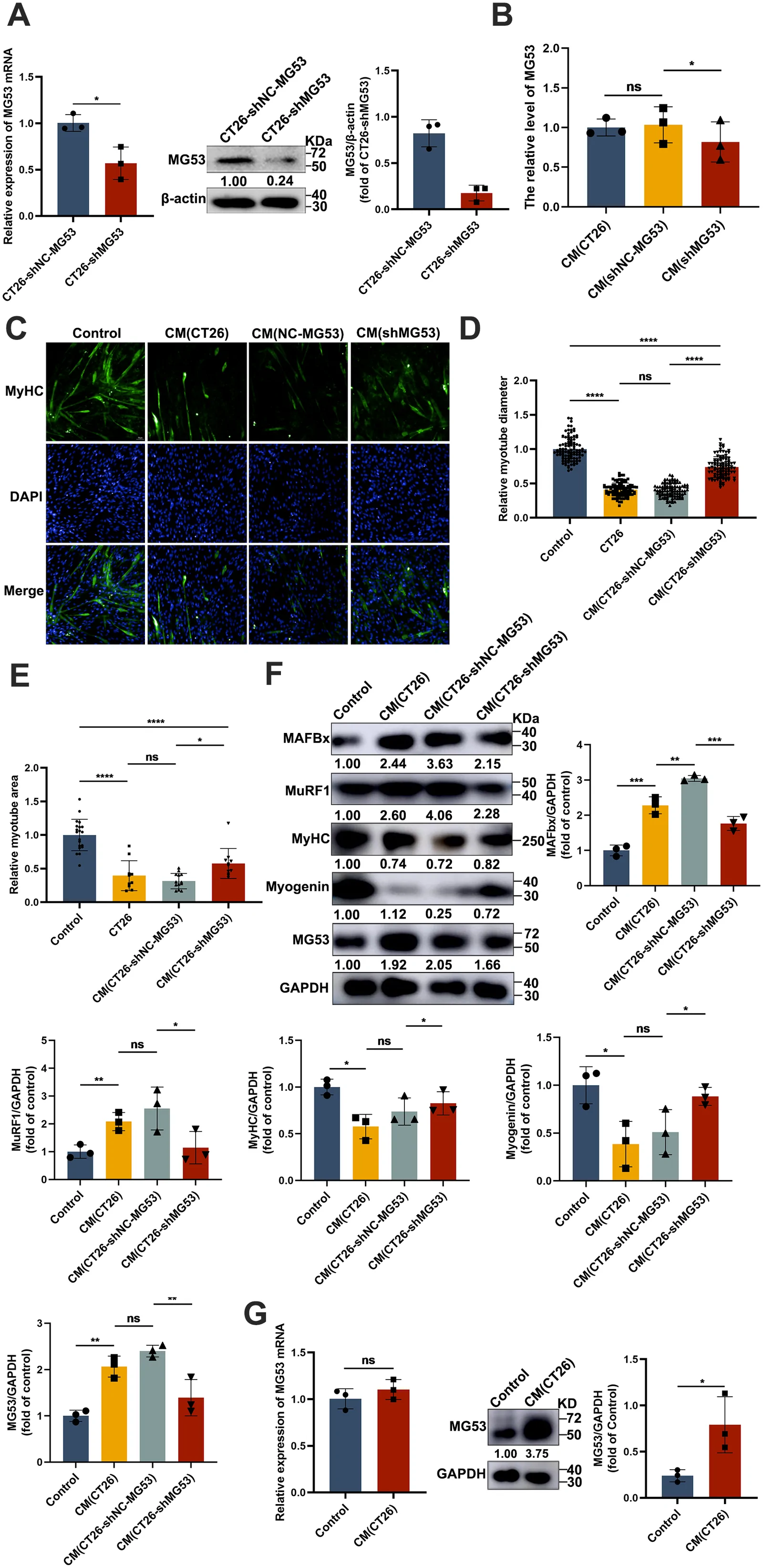
Changes in related protein expression levels in C2C12 myotubes treated with CM (CT26-shMG53) and MyHC immunofluorescence staining of C2C12 myotubes. **(A)** Knockdown efficiency in CT26-shMG53 cells was verified by WB and qRT-PCR experiments. **(B)** MG53 levels in CM (CT26-shNC-MG53) and CM (CT26-shMG53) were detected by ELISA. **(C)** MyHC immunofluorescence staining of C2C12 myotubes. **(D)** Diameter of immunofluorescence-stained myotubes was statistically analyzed using ImageJ software. **(E)** Area of immunofluorescence-stained myotubes was statistically analyzed using ImageJ software. **(F)** Protein expression levels of MG53, Myogenin, MyHC, MuRF1, and MAFbx in myotube cells were detected by WB. Relative quantification of muscle atrophy- and synthesis-related proteins was performed using image J software. **(G)** MG53 protein and RNA levels in myotubes after CM (CT26) treatment were detected by WB and qRT-PCR experiments. Results are expressed as mean ± SD. One-way ANOVA followed by *post-hoc* multiple comparisons test (Tukey’s HSD test) using Prism version 8.0 (GraphPad, CA, USA) was used to analyze the statistical significance of differences between groups. For all analyses, *p < 0.05, **p < 0.01, ***p < 0.001, ****p < 0.0001.

The above experiments demonstrated that MG53 induces myotube atrophy both *in vitro* and *in vivo*. To investigate its role in cancer cachexia-associated muscle atrophy, we generated MG53-knockdown CT26 cell lines (CT26-shNC-MG53 and CT26-shMG53), verified by WB and qRT-PCR (**Figure 4A**). Conditioned medium (CM) was collected from these cells, and ELISA showed reduced MG53 levels in CM (CT26-shMG53) compared to CM (CT26-shNC-MG53) (**Figure 4B**). CM was mixed 1:1 with differentiation medium and applied to day 3 differentiated C2C12 myotubes for 72 h. Immunofluorescence revealed increased myotube diameter and area in the CM (CT26-shMG53) group versus CM (CT26-shNC-MG53) (**Figures 4C–E**). WB analysis of day 6 myotubes showed that CM (CT26-shMG53) treatment reduced MG53, MuRF1, and MAFbx expression, while increasing Myogenin and MyHC compared to CM (CT26-shNC-MG53) (**Figure 4F**). Additionally, treating C2C12 myotubes with CM (CT26) for 72 h increased MG53 protein levels without significant changes in MG53 RNA levels (**Figure 4G**). These results indicate that MG53 in CT26 supernatant contributes to cancer cachexia-associated muscle atrophy.

#### 3.4.2 Mice bearing subcutaneous tumors from MG53-knockdown CT26 cells exhibited alleviated cancer cachexia

**Figure 5:**
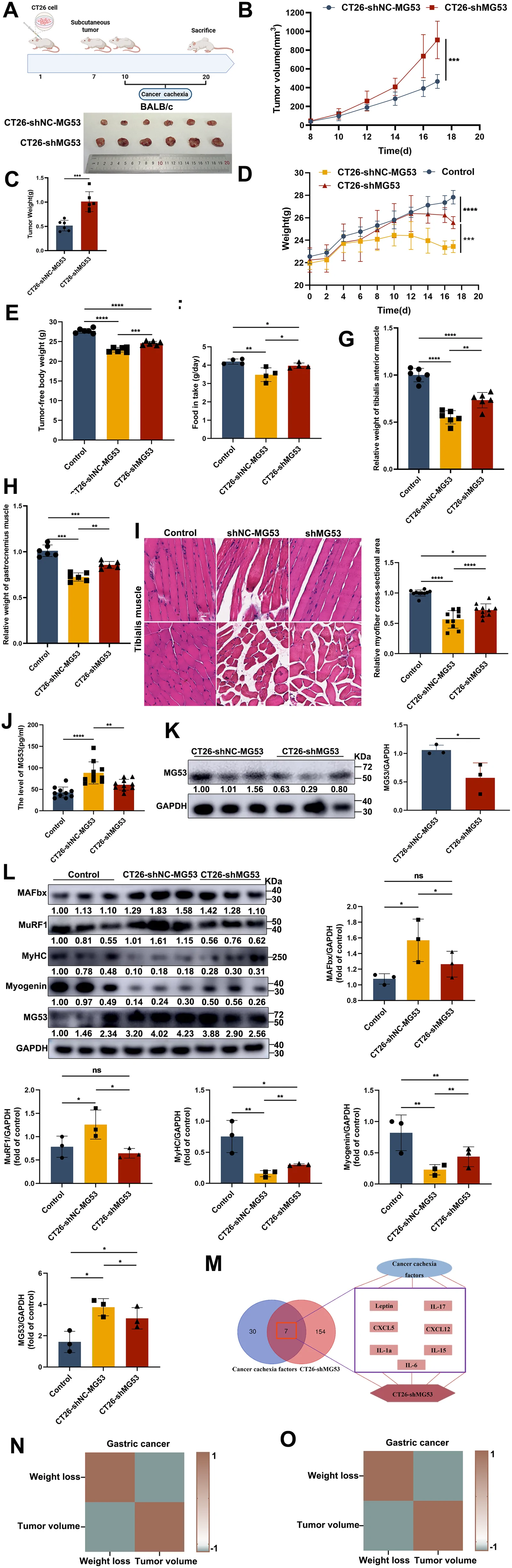
Cancer cachexia symptoms in mice bearing CT26-shMG53 subcutaneous tumors. **(A)** Animal experiment flowchart and subcutaneous tumors from CT26-shMG53 and CT26-shNC-MG53 cells (n=6); **(B)** Growth curve of subcutaneous tumors from CT26-shMG53 and CT26-shNC-MG53 cells (n=6, p < 0.001); **(C)** Tumor weight of subcutaneous tumors from CT26-shMG53 and CT26-shNC-MG53 cells (n=6, p < 0.001); **(D)** Intersection of RNA-Seq results from subcutaneous tumor tissues of CT26-shMG53 and CT26-shNC-MG53 cells with currently known cancer cachexia factors; **(E)** Correlation analysis between tumor volume and degree of weight loss in colorectal cancer patients with cachexia (n=18); **(F)** Correlation analysis between tumor volume and degree of weight loss in gastric cancer patients with cachexia (n=25); **(G)** Body weight change curve of mice bearing subcutaneous tumors from CT26-shMG53 and CT26-shNC-MG53 cells (n=6, p < 0.001); **(H)** Tumor-free body weight of mice bearing subcutaneous tumors from CT26-shMG53 and CT26-shNC-MG53 cells (n=6, p < 0.001); **(I)** Food intake of mice bearing subcutaneous tumors from CT26-shMG53 and CT26-shNC-MG53 cells (n=1, p < 0.05); **(J)** Tibialis anterior muscle weight of mice bearing subcutaneous tumors from CT26-shMG53 and CT26-shNC-MG53 cells (n=6, p < 0.05); **(K)** Gastrocnemius muscle weight of mice bearing subcutaneous tumors from CT26-shMG53 and CT26-shNC-MG53 cells (n=6, p < 0.05); **(L)** HE staining of the tibialis anterior muscle from mice bearing subcutaneous tumors from CT26-shMG53 and CT26-shNC-MG53 cells (n=3); **(M)** Serum MG53 levels in mice bearing subcutaneous tumors from CT26-shMG53 and CT26-shNC-MG53 cells detected by ELISA (n=10); **(N)** MG53 protein expression levels in subcutaneous tumor tissues from mice bearing CT26-shMG53 and CT26-shNC-MG53 tumors detected by WB (n=3). Relative quantification of proteins was performed using Image J software; **(O)** Protein expression levels of MG53, Myogenin, MyHC, MuRF1, and MAFbx in the tibialis anterior muscle of mice bearing subcutaneous tumors from CT26-shMG53 and CT26-shNC-MG53 cells detected by WB (n=3). Relative quantification of muscle atrophy- and synthesis-related proteins was performed using image J software. Results are expressed as mean ± SD. One-way ANOVA followed by *post-hoc* multiple comparisons test (Tukey’s HSD test) using Prism version 8.0 (GraphPad, CA, USA) was used to analyze the statistical significance of differences between groups. For all analyses, *p < 0.05, **p < 0.01, ***p < 0.001, ****p < 0.0001.

Subsequently, CT26-shNC-MG53 and CT26-shMG53 cells were subcutaneously inoculated into the right dorsal flank of BALB/c mice. Palpable tumors were detected on day 7 (**Figure 5A**). Body weight, food intake, and tumor size were measured every two days. Mice were euthanized on day 17 (Note: the 20 days indicated in the animal model schematic represents an approximate duration; the exact timing varies slightly depending on the subcutaneous tumor cell type used). Tumors, serum, and muscle tissues were collected. CT26-shMG53 tumors were larger in volume (**Figures 5A, B**) and heavier (**Figure 5C**) than CT26-shNC-MG53 tumors. Subcutaneous tumors, serum, and muscle tissues were collected. However, mice bearing CT26-shMG53 tumors showed attenuated body weight loss (**Figure 5D**), increased tumor-free body weight (**Figure 5E**), and improved food intake (**Figure 5F**). Tibialis anterior and gastrocnemius muscle weights were significantly increased in the CT26-shMG53 group (**Figures 5G, H**). HE staining of TA muscle showed reduced fiber disorganization and increased diameter in CT26-shMG53 tumor-bearing mice (**Figure 5I**), with similar findings in gastrocnemius (**Supplementary Figure 2D**). WB analysis revealed decreased MG53 levels in CT26-shMG53 tumors (**Figure 5J**), and ELISA showed significantly reduced serum MG53 in these mice (**Figure 5K**). WB of TA muscle demonstrated decreased MG53, MuRF1, and MAFbx, and increased Myogenin and MyHC in the CT26-shMG53 group (**Figure 5L**), with consistent results in gastrocnemius (**Supplementary Figure 4A**). These results indicate that although subcutaneous tumors from MG53-knockdown CT26 cells exhibited increased volume and weight, the mice bearing these tumors showed improvesd cancer cachexia symptoms, including reduced body weight loss, attenuated muscle atrophy, and improved anorexia, compared to mice bearing CT26-shNC-MG53 tumors. This phenomenon correlates with lower MG53 levels within the tumors, reduced MG53 release into the bloodstream, and consequently, attenuated effects on muscle tissue. This presents a paradox where tumor growth increases while cachexia symptoms are improvesd. To explain this, we performed RNA-Seq analysis on subcutaneous tumors from CT26-shMG53 and CT26-shNC-MG53 groups. Intersecting the differentially expressed genes from the sequencing results with previously summarized cancer cachexia factors from our published work (8), we found that known cachexia-inducing factors, such as leptin, IL-17, IL-1a, IL-15, IL-6, CXCL5, and CXCL12, were significantly reduced in CT26-shMG53 tumors compared to CT26-shNC-MG53 tumors (**Figure 5M**). Additionally, we investigated whether tumor size correlates with the degree of weight loss in clinical cancer cachexia patients. By analyzing CT images and pathological reports to determine tumor volume, the study revealed no significant correlation between tumor volume and the extent of weight loss in colorectal and gastric cancer patients with cachexia (**Figures 5N, O**). Notably, previous studies have suggested an anti-tumor role for MG53 in colon cancer (16, 40), which is consistent with our observation that tumor size increased after MG53 knockdown in CT26 cells. Furthermore, it is clinically observed that tumor size does not necessarily correlate with cachexia severity; for example, pancreatic cancer can cause severe cachexia even with small tumors (41, 42). This phenomenon may be explained by the fact that dying tumor cells release inflammatory factors that drive cachexia (43). In gastrointestinal tumors, increased cell death within the tumor mass can trigger the release of pro-inflammatory cytokines, thereby exacerbating systemic inflammation and cachexia regardless of overall tumor size. Conversely, reduced tumor cell death may lead to lower levels of such inflammatory mediators, which could partly explain why cachexia symptoms were improved in our model despite increased tumor size. This finding may partly explain why cachexia symptoms were improved despite increased tumor size.

#### 3.4.3 Atrophy of C2C12 myotubes induced by supernatant from MG53-overexpressing CT26 cells is aggravated

**Figure 6:**
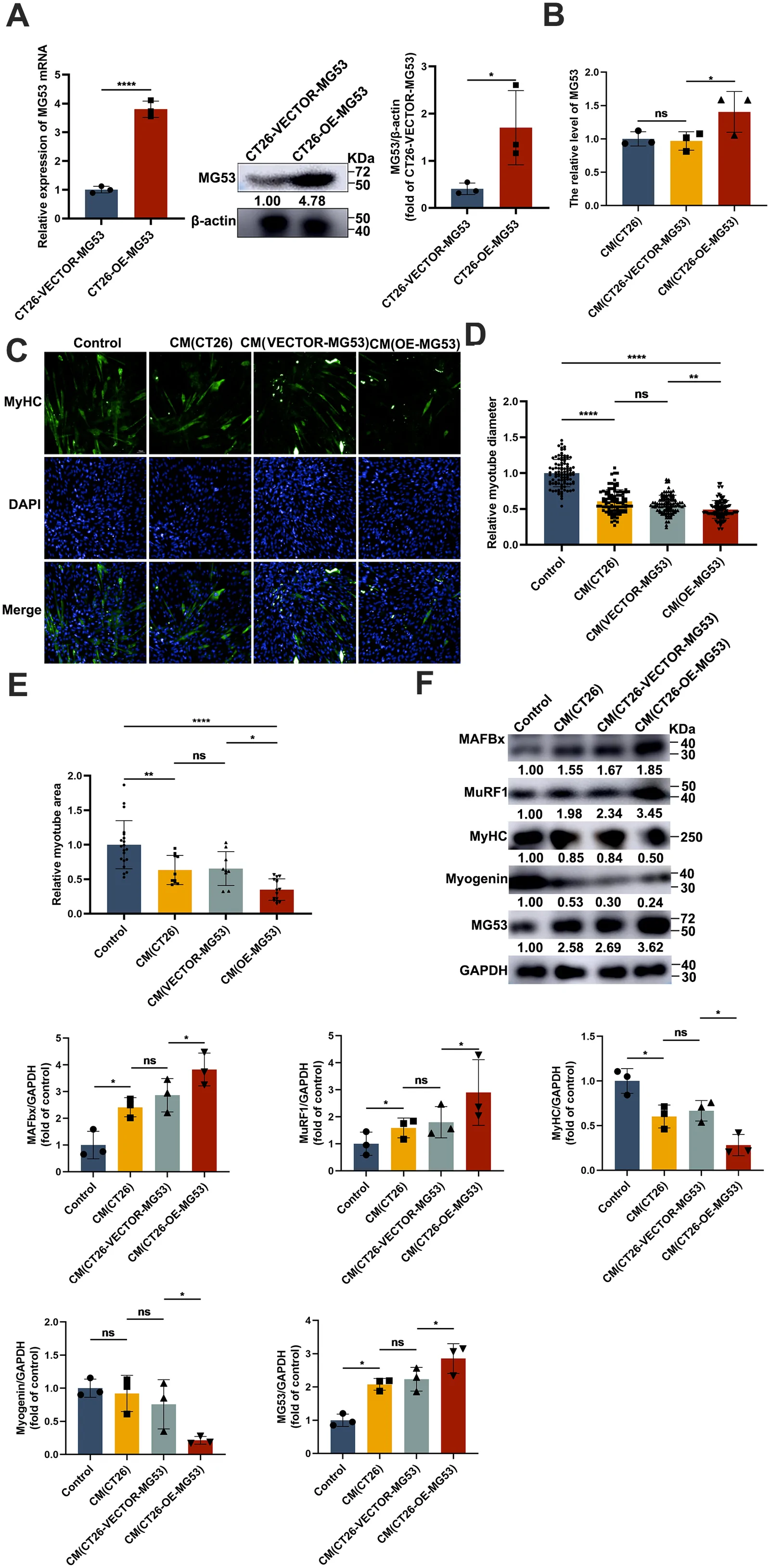
Changes in related protein expression levels in C2C12 myotubes treated with CM (CT26-OE-MG53) and MyHC immunofluorescence staining of C2C12 myotubes. **(A)** Overexpression efficiency in CT26-OE-MG53 cells was verified by WB and qRT-PCR experiments; **(B)** MG53 levels in CM (CT26-VECTOR-MG53) and CM(CT26-OE-MG53) were detected by ELISA; **(C)** MyHC immunofluorescence staining of C2C12 myotubes; **(D)** Diameter of immunofluorescence-stained myotubes was statistically analyzed using Image J software; **(E)** Area of immunofluorescence-stained myotubes was statistically analyzed using Image J software; **(F)** Protein expression levels of MG53, Myogenin, MyHC, MuRF1, and MAFbx in myotube cells were detected by WB. Relative quantification of muscle atrophy- and synthesis-related proteins was performed using ImageJ software. Results are expressed as mean ± SD. One-way ANOVA followed by *post-hoc* multiple comparisons test (Tukey’s HSD test) using Prism version 8.0 (GraphPad, CA, USA) was used to analyze the statistical significance of differences between groups. For all analyses, *p < 0.05, **p < 0.01, ***p < 0.001, ****p < 0.0001.

To further investigate the role of MG53 in cancer cachexia, we generated two stable MG53-overexpressing CT26 cell lines, CT26-VECTOR-MG53 and CT26-OE-MG53, and confirmed their overexpression efficiency by Western blotting and qRT-PCR (**Figure 6A**). We then collected the supernatant from CT26 -VECTOR-MG53 and CT26-OE-MG53 cells as cancer cachexia conditioned medium (CM), named CM (CT26-VECTOR-MG53) and CM (CT26-OE-MG53), respectively. Using a mouse MG53 ELISA kit, we detected MG53 levels in CM (CT26-VECTOR-MG53) and CM (CT26-OE-MG53) and found that the MG53 level in CM (CT26-OE-MG53) was increased compared to CM (CT26-VECTOR-MG53) (**Figure 6B**). The conditioned medium was then mixed 1:1 with freshly prepared horse serum differentiation medium and applied to day 3 differentiated C2C12 myotubes for 72 hours. Immunofluorescence was performed, and myotube diameter and area were measured and statistically analyzed using image J software. We observed that the myotube diameter and area in the CM (CT26-OE-MG53) group were decreased compared to the CM (CT26-VECTOR-MG53) group (**Figures 6C–E**). Subsequently, we performed WB on day 6 differentiated C2C12 myotubes treated with CM (CT26-VECTOR-MG53) or CM (CT26-OE-MG53). Compared to CM (CT26-VECTOR-MG53) treatment, CM (CT26-OE-MG53) treatment resulted in increased MG53 protein expression levels, significantly increased expression of muscle atrophy-related proteins MuRF1 and MAFbx, and significantly decreased expression of myotube synthesis-related proteins Myogenin and MyHC in C2C12 myotubes (**Figure 6F**). These experiments indicate that the supernatant from MG53-overexpressing CT26 cells exacerbates cancer cachexia-associated muscle atrophy.

#### 3.4.4 Mice bearing subcutaneous tumors from MG53-overexpressing CT26 cells exhibited aggravated cancer cachexia symptoms

**Figure 7:**
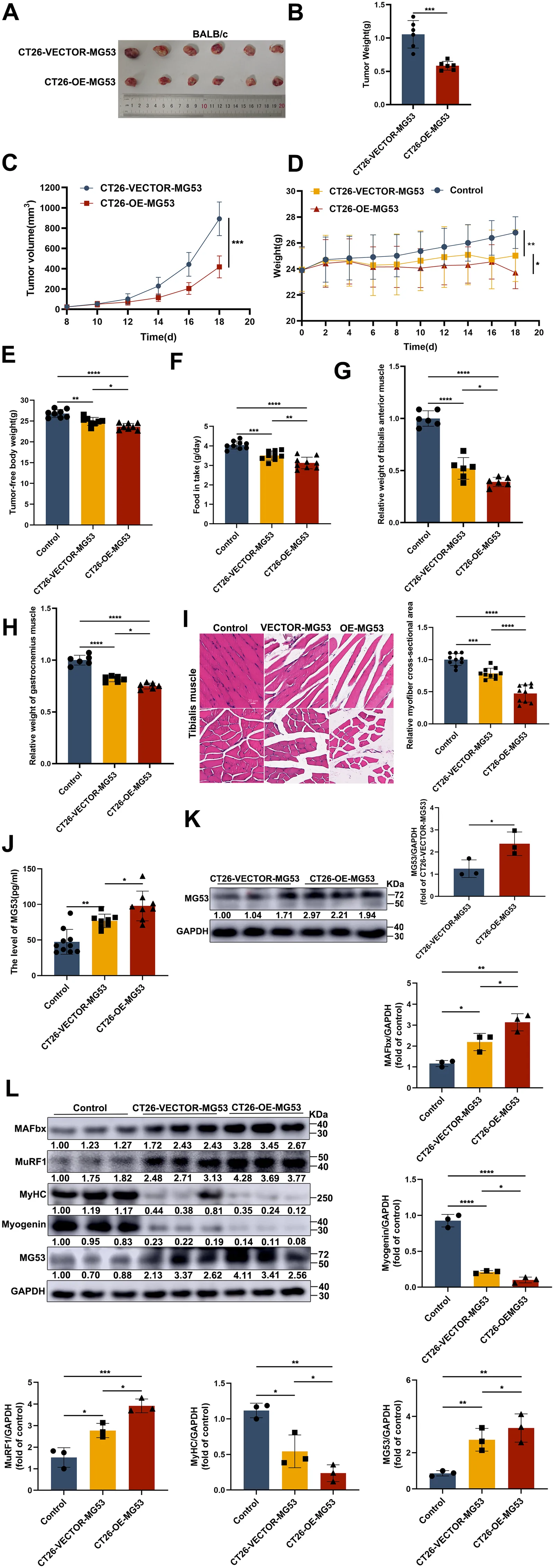
Cancer cachexia symptoms in mice bearing CT26-OE-MG53 subcutaneous tumors. **(A)** Subcutaneous tumors from CT26-OE-MG53 and CT26-VECTOR-MG53 cells (n=6); **(B)** Tumor weight of subcutaneous tumors from CT26-OE-MG53 and CT26-VECTOR-MG53 cells (n=6, p < 0.001); **(C)** Growth curve of subcutaneous tumors from CT26-OE-MG53 and CT26-VECTOR-MG53 cells (n=6, p < 0.001); **(D)** Body weight change curve of mice bearing subcutaneous tumors from CT26-OE-MG53 and CT26-VECTOR-MG53 cells (n=10, p < 0.05); **(E)** Tumor-free body weight of mice bearing subcutaneous tumors from CT26-OE-MG53 and CT26-VECTOR-MG53 cells (n=8, p < 0.05); **(F)** Food intake of mice bearing subcutaneous tumors from CT26-OE-MG53 and CT26-VECTOR-MG53 cells (n=10, p < 0.01); **(G)** Tibialis anterior muscle weight of mice bearing subcutaneous tumors from CT26-OE-MG53 and CT26-VECTOR-MG53 cells (n=6, p < 0.05); **(H)** Gastrocnemius muscle weight of mice bearing subcutaneous tumors from CT26-OE-MG53 and CT26-VECTOR-MG53 cells (n=6, p < 0.01); **(I)** HE staining of the tibialis anterior muscle from mice bearing subcutaneous tumors from CT26-OE-MG53 and CT26-VECTOR-MG53 cells (n=3); **(J)** Serum MG53 levels in mice bearing subcutaneous tumors from CT26-OE-MG53 and CT26-VECTOR-MG53 cells detected by ELISA (n=10, p < 0.05); **(K)** MG53 protein expression levels in subcutaneous tumor tissues from mice bearing CT26-OE-MG53 and CT26-VECTOR-MG53 tumors detected by WB (n=3). Relative quantification of proteins was performed using ImageJ software; **(L)** Protein expression levels of MG53, Myogenin, MyHC, MuRF1, and MAFbx in the tibialis anterior muscle of mice bearing subcutaneous tumors from CT26-OE-MG53 and CT26-VECTOR-MG53 cells detected by WB (n=3). Relative quantification of muscle atrophy- and synthesis-related proteins was performed using Image J software. Results are expressed as mean ± SD. One-way ANOVA followed by *post-hoc* multiple comparisons test (Tukey’s HSD test) using Prism version 8.0 (GraphPad, CA, USA) was used to analyze the statistical significance of differences between groups. For all analyses, *p < 0.05, **p < 0.01, ***p < 0.001, ****p < 0.0001.

Subsequently, we subcutaneously inoculated CT26-VECTOR-MG53 and CT26-OE-MG53 cells into the right dorsal flank of BALB/c mice. Subcutaneous tumors are shown in **Figure 7A**. Body weight, food intake, and tumor size were measured every two days. Mice were euthanized on day 18, and subcutaneous tumors, serum, and muscle tissues were collected. We found that subcutaneous tumors from CT26-OE-MG53 cells exhibited reduced volume (**Figure 7C**) and decreased weight (**Figure 7B**) compared to those from CT26-VECTOR-MG53 cells. However, mice bearing CT26-OE-MG53 tumors demonstrated exacerbated body weight loss (**Figure 7D**), reduced tumor-free body weight (**Figure 7E**), and decreased food intake (**Figure 7F**) compared to mice bearing CT26-VECTOR-MG53 tumors. Additionally, the weights of the tibialis anterior and gastrocnemius muscles were significantly reduced in mice bearing CT26-OE-MG53 tumors compared to those bearing CT26-VECTOR-MG53 tumors (**Figures 7G, H**). HE staining of the tibialis anterior muscle revealed increased muscle fiber disorganization and reduced myofiber diameter in mice bearing CT26-OE-MG53 tumors compared to those bearing CT26-VECTOR-MG53 tumors (**Figure 7I**). Furthermore, HE staining of the gastrocnemius muscle revealed disordered arrangement and reduced diameter of muscle fibers in subcutaneous tumor-bearing mice with MG53-overexpressing CT26 cells (**Supplementary Figure 2E**). WB analysis of subcutaneous tumor tissues showed increased MG53 levels in tumors from CT26-OE-MG53 tumor-bearing mice compared to those from CT26-VECTOR-MG53 tumor-bearing mice (**Figure 7K**). ELISA results indicated significantly elevated serum MG53 levels in CT26-OE-MG53 tumor-bearing mice compared to CT26-VECTOR-MG53 tumor-bearing mice (**Figure 7J**). WB analysis of the tibialis anterior muscle demonstrated increased MG53 protein expression, significantly elevated levels of muscle atrophy-related proteins MuRF1 and MAFbx, and significantly decreased levels of muscle synthesis-related proteins Myogenin and MyHC in muscles from CT26-OE-MG53 tumor-bearing mice compared to those from CT26-VECTOR-MG53 tumor-bearing mice (**Figure 7L**). Consistent findings were also observed by WB analysis of gastrocnemius muscle tissue (**Supplementary Figure 4C**). These results indicate that although subcutaneous tumors from MG53-overexpressing CT26 cells exhibited reduced volume and weight, the mice bearing these tumors showed aggravated cancer cachexia symptoms, including increased body weight loss, exacerbated muscle wasting, and worsened anorexia, compared to mice bearing CT26-VECTOR-MG53 tumors.

#### 3.4.5 Supernatant from MG53-overexpressing MC38 cells induces atrophy of C2C12 myotubes

**Figure 8:**
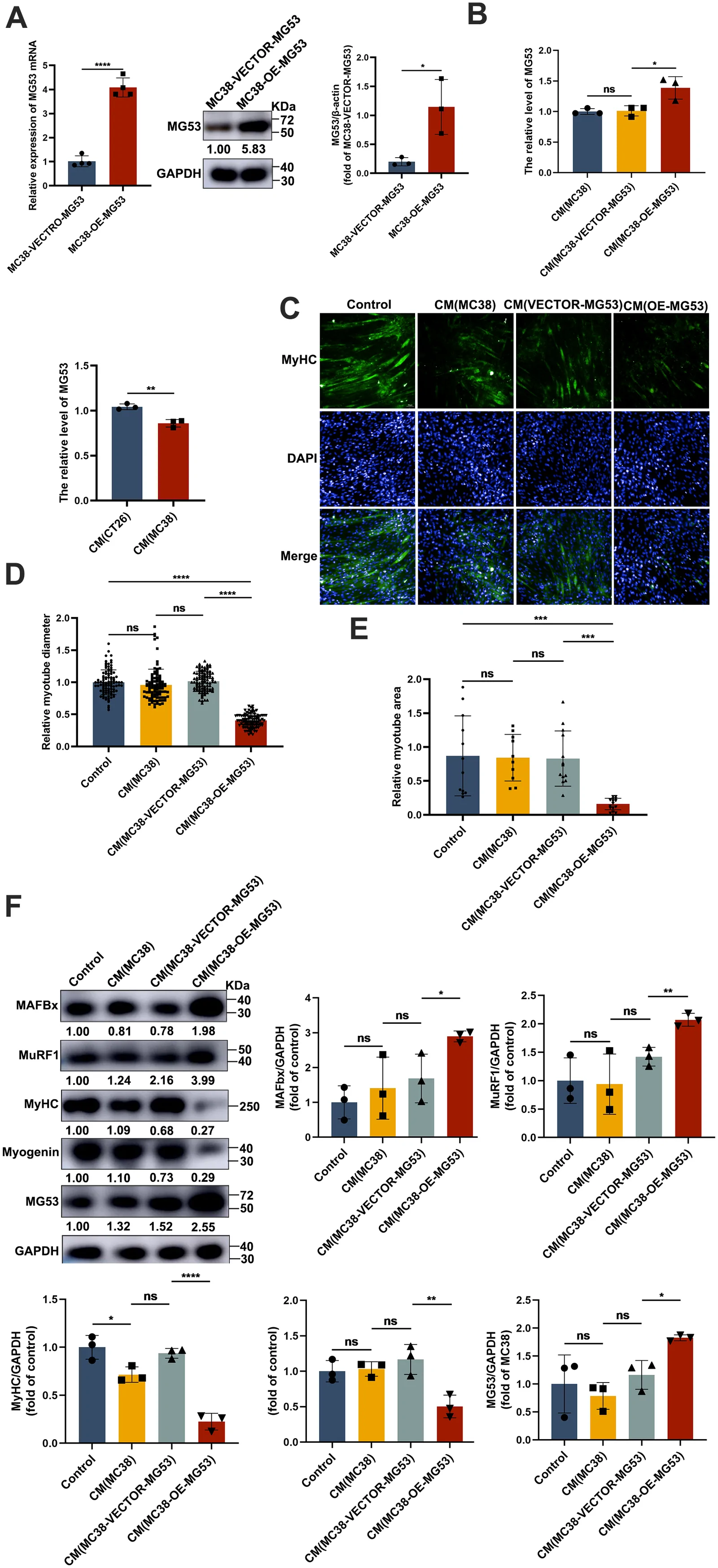
Changes in expression levels of related proteins in C2C12 myotubes treated with CM (MC38-OE-MG53) and MyHC immunofluorescence staining of C2C12 myotubes. **(A)** Overexpression efficiency in MC38-OE-MG53 cells was verified by WB and qRT-PCR experiments. **(B)** MG53 levels in CM (MC38-VECTOR-MG53), CM (MC38-OE-MG53), CM (CT26) and CM (MC38) were detected by ELISA (p<0.05). **(C)** MyHC immunofluorescence staining of C2C12 myotubes. **(D)** Diameter of immunofluorescence-stained myotubes was statistically analyzed using ImageJ software. **(E)** Area of immunofluorescence-stained myotubes was statistically analyzed using ImageJ software. **(F)** Protein expression levels of MG53, Myogenin, MyHC, MuRF1, and MAFbx in myotube cells were detected by WB. Relative quantification of muscle atrophy- and synthesis-related proteins was performed using ImageJ software. Results are expressed as mean ± SD. One-way ANOVA followed by *post-hoc* multiple comparisons test (Tukey’s HSD test) using Prism version 8.0 (GraphPad, CA, USA) was used to analyze the statistical significance of differences between groups. For all analyses, *p < 0.05, **p < 0.01, ***p < 0.001, ****p < 0.0001.

Previous studies have indicated that MC38 cells do not induce cancer cachexia and are often used as a control for CT26 in cancer cachexia research (44). To further investigate the role of MG53, we constructed MG53-overexpressing MC38 cell lines, MC38-VECTOR-MG53 and MC38-OE-MG53, with overexpression efficiency verified by WB and qRT-PCR (**Figure 8A**). We then collected the supernatant from MC38-VECTOR-MG53 and MC38-OE-MG53 cells as cancer cachexia conditioned medium (CM), named CM (MC38-VECTOR-MG53) and CM (MC38-OE-MG53), respectively. MG53 levels were measured by ELISA, showing increased levels in CM (MC38-OE-MG53) versus CM (MC38-VECTOR-MG53), and significantly lower levels in CM (MC38) compared to CM (CT26) (**Figure 8B**). CM was mixed 1:1 with differentiation medium and applied to day 3 differentiated C2C12 myotubes for 72 h. Immunofluorescence analysis revealed reduced myotube diameter and area in the CM (MC38-OE-MG53) group compared to the CM (MC38-VECTOR-MG53) group, with no significant change versus control (**Figures 8C–E**). Subsequently, WB analysis of day 6 myotubes treated with CM (MC38-OE-MG53) showed increased MG53, MuRF1, and MAFbx, and decreased Myogenin and MyHC compared to CM (MC38-VECTOR-MG53), with no significant changes versus control (**Figure 8F**). These results indicate that supernatant from MG53-overexpressing MC38 cells induces muscle atrophy. Consistent with previous findings, our study confirmed that MC38 cells do not induce cancer cachexia-related muscle atrophy compared to CT26 cells (45).

#### 3.4.6 Mice bearing subcutaneous tumors from MG53-overexpressing MC38 cells developed cancer cachexia symptoms

**Figure 9:**
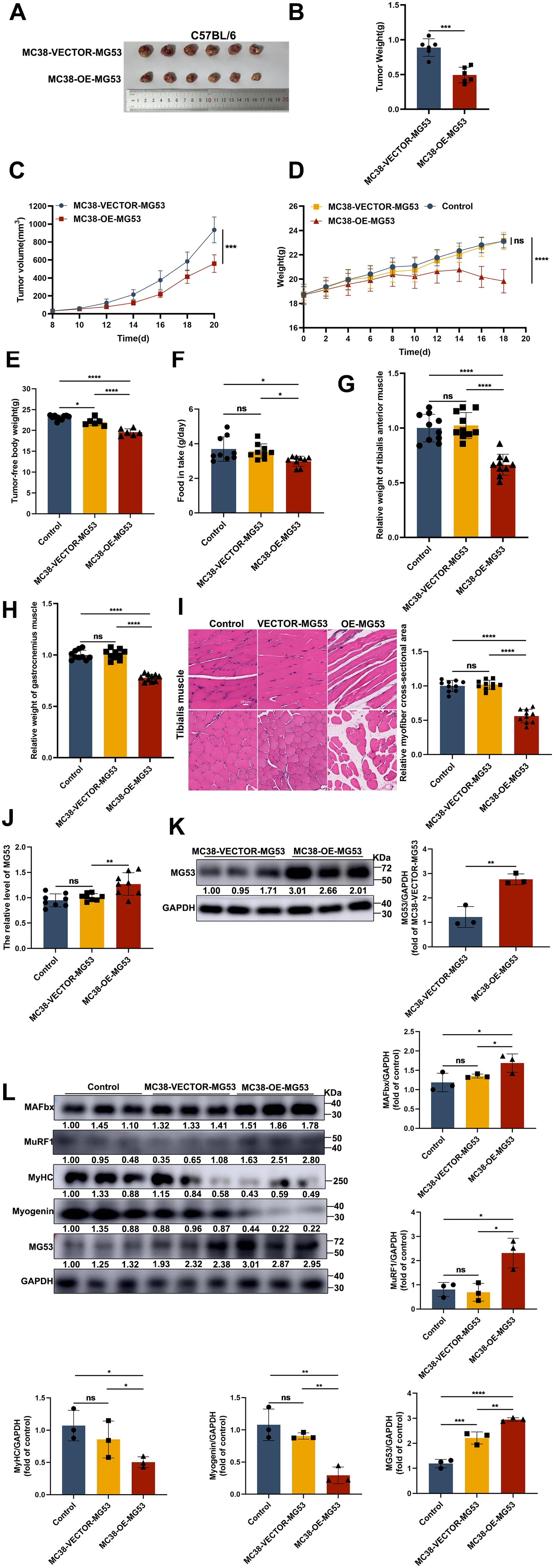
Cancer cachexia symptoms in mice bearing MC38-OE-MG53 subcutaneous tumors. **(A)** Subcutaneous tumors from MC38-OE-MG53 and MC38-VECTOR-MG53 cells (n=6); **(B)** Tumor weight of subcutaneous tumors from MC38-OE-MG53 and MC38-VECTOR-MG53 cells (n=6, p < 0.001); **(C)** Growth curve of subcutaneous tumors from MC38-OE-MG53 and MC38-VECTOR-MG53 cells (n=6, p < 0.001); **(D)** Body weight change curve of mice bearing subcutaneous tumors from MC38-OE-MG53 and MC38-VECTOR-MG53 cells (n=6, p < 0.0001); **(E)** Tumor-free body weight of mice bearing subcutaneous tumors from MC38-OE-MG53 and MC38-VECTOR-MG53 cells (n=6, p < 0.0001); **(F)** Food intake of mice bearing subcutaneous tumors from MC38-OE-MG53 and MC38-VECTOR-MG53 cells (n=10, p < 0.05); **(G)** Tibialis anterior muscle weight of mice bearing subcutaneous tumors from MC38-OE-MG53 and MC38-VECTOR-MG53 cells (n=10, p < 0.001); **(H)** Gastrocnemius muscle weight of mice bearing subcutaneous tumors from MC38-OE-MG53 and MC38-VECTOR-MG53 cells (n=10, p < 0.01); **(I)** HE staining of the tibialis anterior muscle from mice bearing subcutaneous tumors from MC38-OE-MG53 and MC38-VECTOR-MG53 cells (n=3); **(J)** Serum MG53 levels in mice bearing subcutaneous tumors from MC38-OE-MG53 and MC38-VECTOR-MG53 cells detected by ELISA (n=8, p < 0.01); **(K)** MG53 protein expression levels in subcutaneous tumor tissues from mice bearing MC38-OE-MG53 and MC38-VECTOR-MG53 tumors detected by WB (n=3). Relative quantification of proteins was performed using ImageJ software; **(L)** Protein expression levels of MG53, Myogenin, MyHC, MuRF1, and MAFbx in the tibialis anterior muscle of mice bearing subcutaneous tumors from MC38-OE-MG53 and MC38-VECTOR-MG53 cells detected by WB (n=3). Relative quantification of muscle atrophy- and synthesis-related proteins was performed using Image J software. Results are expressed as mean ± SD. One-way ANOVA followed by *post-hoc* multiple comparisons test (Tukey’s HSD test) using Prism version 8.0 (GraphPad, CA, USA) was used to analyze the statistical significance of differences between groups. For all analyses, *p < 0.05, **p < 0.01, ***p < 0.001, ****p < 0.0001.

Subsequently, MC38-VECTOR-MG53 and MC38-OE-MG53 cells were subcutaneously inoculated into the right dorsal flank of C57BL/6 mice (**Figure 9A**). Body weight, food intake, and tumor size were measured every two days. Mice were euthanized on day 20, and tumors, serum, and muscle tissues were collected. Compared to MC38-VECTOR-MG53 tumors, MC38-OE-MG53 tumors had reduced weight (**Figure 9B**) and volume (**Figure 9C**). However, mice bearing MC38-OE-MG53 tumors showed exacerbated body weight loss (**Figure 9D**), reduced tumor-free body weight (**Figure 9E**), and decreased food intake, whereas no significant food intake changes were observed in MC38-VECTOR-MG53 tumor-bearing mice versus controls (**Figure 9F**). Tibialis anterior and gastrocnemius muscle weights were significantly reduced in MC38-OE-MG53 tumor-bearing mice compared to MC38-VECTOR-MG53 group, with no significant changes in the latter versus controls (**Figures 9G, H**). HE staining of tibialis anterior and gastrocnemius muscles revealed increased fiber disorganization and reduced diameter in MC38-OE-MG53 tumor-bearing mice, while no changes were seen in MC38-VECTOR-MG53 mice versus controls (**Figure 9I**, **Supplementary Figure 2F**). WB analysis showed increased MG53 levels in MC38-OE-MG53 tumors (**Figure 9K**) and gastrocnemius muscle (**Supplementary Figure 4E**), with no changes in MC38-VECTOR-MG53 versus controls. ELISA revealed significantly elevated serum MG53 in MC38-OE-MG53 tumor-bearing mice, with no changes in MC38-VECTOR-MG53 versus controls (**Figure 9J**). WB of tibialis anterior muscle showed increased MG53, MuRF1, and MAFbx, and decreased Myogenin and MyHC in MC38-OE-MG53 tumor-bearing mice, with no significant changes in MC38-VECTOR-MG53 versus controls (**Figure 9L**). These results indicate that although MC38-OE-MG53 tumors were smaller, they aggravated cancer cachexia symptoms, including body weight loss, muscle wasting, and anorexia, compared to MC38-VECTOR-MG53 tumors.

### 3.5 Tumor-derived MG53 aggravates cancer cachexia-related muscle atrophy by inhibiting the TLR4/PI3K/AKT/FOXO3a signaling pathway

**Figure 10:**
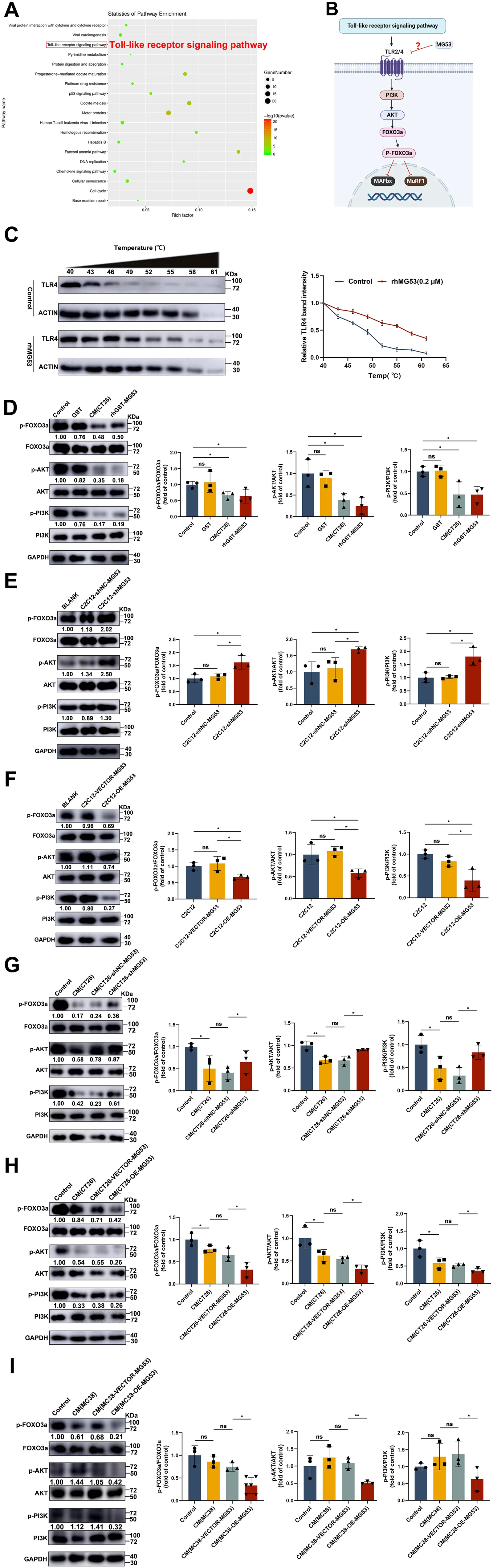
MG53 aggravates cancer cachexia muscle atrophy by inhibiting the TLR4/PI3K/AKT/FOXO3a signaling pathway. **(A)** RNA-Seq was performed on C2C12 myotubes treated with rhMG53, C2C12 (shMG53), and C2C12 myotubes on day 6 of differentiation treated with CM (CT26-shMG53). KEGG pathway enrichment analysis revealed the main upregulated pathways related to muscle metabolism enriched in C2C12 myotubes treated with CM (CT26-shMG53); **(B)** Hypothesized signaling mechanism of MG53-induced muscle atrophy; **(C)** Thermal stability assy investigating the interaction between MG53 and TLR4; **(D)** WB analysis of changes in PI3K, AKT, and FOXO3a protein expression levels in rhMG53-treated C2C12 myotubes; **(E, F)**: WB analysis of changes in PI3K, AKT, and FOXO3a protein expression levels in C2C12 -shMG53 and C2C12-OE-MG53 myotubes; **(G–I)** WB analysis of changes in PI3K, AKT, and FOXO3a protein expression levels in C2C12 myotubes treated with CM(CT26-shMG53), CM(CT26-OE-MG53), and CM(MC38-OE-MG53). Relative quantification of each phosphorylated form to its corresponding inactive form was performed using Image J software. Results are expressed as mean ± SD. One-way ANOVA followed by post-hoc multiple comparisons test (Tukey's HSD test) using Prism version 8.0 (GraphPad, CA, USA) was used to analyze the statistical significance of differences between groups. For all analyses, * *p* < 0.05, ** *p* < 0.01, *** *p* < 0.001, **** *p* < 0.0001.

To investigate the signaling pathway by which MG53 induces cancer cachexia-induced myotube atrophy, we applied CM from CT26-shMG53 and CT26-shNC-MG53 to C2C12 myotubes at day 6 of differentiation and performed RNA-Seq. KEGG enrichment analysis showed that upregulated pathways in CM (CT26-shMG53)-treated myotubes were primarily enriched in the Toll-like receptor signaling pathway (**Figure 10A**). Previous studies have shown that TLR4 is involved in hippocampal neuronal apoptosis via the PI3K/AKT/FOXO3a/Bim pathway (46). Additionally, the PI3K/AKT/FOXO3a pathway is closely linked to muscle metabolism (47), and FOXO3a regulates MuRF1 and MAFbx expression (47, 48). We thus hypothesized that MG53-induced muscle atrophy may involve the Toll-like receptor pathway (**Figure 10B**), and that MG53 suppresses TLR4/PI3K/AKT/FOXO3a signaling, leading to increased MuRF1 and MAFbx expression and subsequent muscle atrophy.

**Figure 11:**
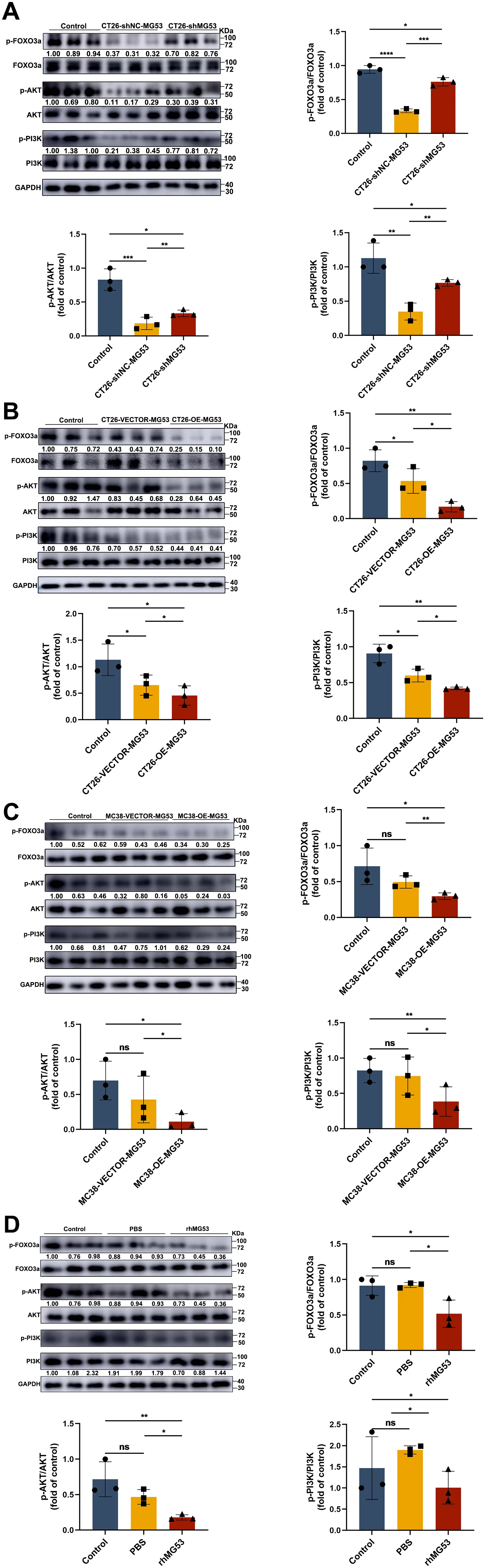
MG53 aggravates cancer cachexia muscle atrophy by inhibiting the TLR4/PI3K/AKT/FOXO3a signaling pathway (western blot validation of muscle tissue proteins). **(A–C)** WB analysis of changes in PI3K, AKT, and FOXO3a protein expression levels in tibialis anterior muscle tissue from subcutaneous tumor-bearing mice of CT26-shMG53, CT26-OE-MG53, and MC38-OE-MG53. **(D)** WB analysis of changes in PI3K, AKT, and FOXO3a protein expression levels in tibialis anterior muscle tissue from BALB/c mice intraperitoneally injected with rhMG53 protein. Relative quantification of each phosphorylated form to its corresponding inactive form was performed using Image J software. Results are expressed as mean ± SD. One-way ANOVA followed by post-hoc multiple comparisons test (Tukey's HSD test) using Prism version 8.0 (GraphPad, CA, USA) was used to analyze the statistical significance of differences between groups. For all analyses, * *p* < 0.05, ** p*p* < 0.01, *** *p* < 0.001, **** *p* < 0.0001.

To validate our hypothesis, C2C12 myotubes were treated with rhMG53 or PBS. Thermal stability assays showed that rhMG53 slowed TLR4 protein degradation compared to controls (**Figure 10C**). C2C12 myotubes were then treated with rhMG53, various conditioned media from CT26 and MC38 cell lines. WB analysis showed that rhMG53 significantly reduced phosphorylation of PI3K, AKT, and FOXO3a (**Figure 10D**). Phosphorylation of these proteins was increased in C2C12-shMG53 myotubes and decreased in C2C12-OE-MG53 myotubes (**Figures 10E, F**). Compared to CM from CT26-shNC-MG53, CM from CT26-shMG53 increased phosphorylation of PI3K, AKT, and FOXO3a, while CM from CT26-OE-MG53 and MC38-OE-MG53 decreased phosphorylation compared to their respective controls (**Figures 10G–I**). WB analysis of tibialis anterior muscle from subcutaneous tumor-bearing mice showed increased phosphorylation of PI3K, AKT, and FOXO3a in CT26-shMG53 mice versus CT26-shNC-MG53, and decreased phosphorylation in CT26-OE-MG53 and MC38-OE-MG53 mice compared to their controls (**Figures 11A–C**). Similarly, intraperitoneal rhMG53 reduced phosphorylation of these proteins in BALB/c tibialis anterior (**Figure 11D**) and in gastrocnemius muscle from CT26-shMG53, CT26-OE-MG53, and MC38-OE-MG53 tumor-bearing mice (**Supplementary Figures 4B, D, F**). WB analysis of tibialis anterior and gastrocnemius muscles from BALB/c (**Supplementary Figure 1B**) and C57BL/6 mice (**Supplementary Figures 1D, F**) intraperitoneally injected with rhMG53 also showed decreased phosphorylation of PI3K, AKT, and FOXO3a in the treated groups.

**Figure 12:**
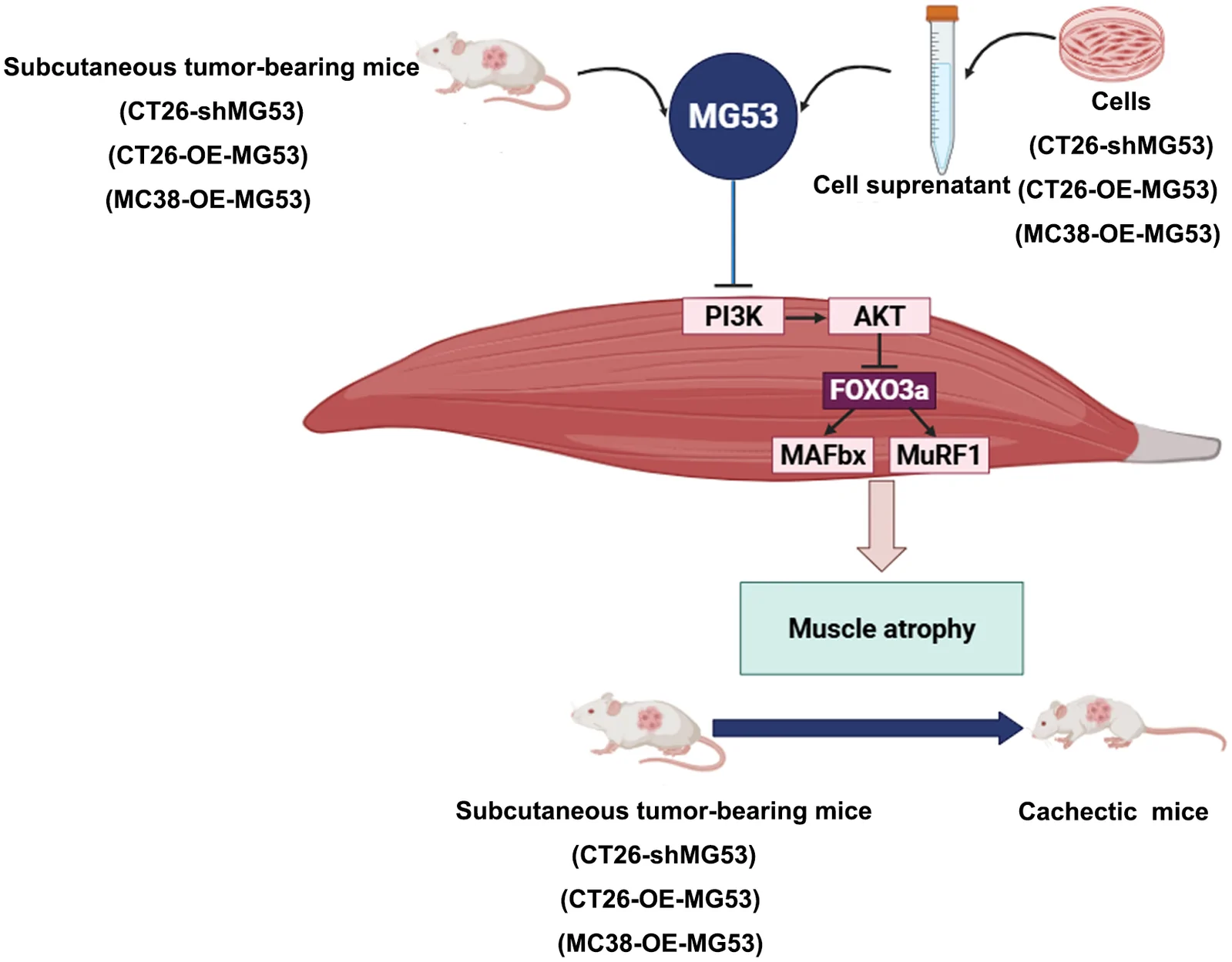
Schematic diagram of the mechanism by which MG53 aggravates cancer cachexia related-muscle atrophy. Tumor-derived MG53 (from tumor cell supernatant or subcutaneous tumors) inhibits the TLR4/PI3K/AKT/FOXO3a pathway, leading to increased expression of muscle atrophy-related proteins MuRF1 and MAFbx in muscle, ultimately resulting in cancer cachexia-related muscle atrophy.

Our above experiments suggest that tumor-derived MG53 can inhibit the TLR4/PI3K/AKT/FOXO3a signaling pathway, leading to increased expression of MuRF1 and MAFbx proteins in muscle, ultimately aggravates cancer cachexia-related muscle atrophy (**Figure 12**).

## 4 Discussion

Our study demonstrates that MG53 exerts antitumor effects (**Supplementary Figures 5, 6**), consistent with previous reports (16, 40, 49–54), while also promoting muscle atrophy in the context of cancer cachexia. This dual role distinguishes MG53 from other known cachectic factors. For instance, IL-6 and TNF-α are well-established pro-inflammatory cytokines that contribute to cancer cachexia, yet clinical and preclinical studies have shown that targeting these factors alone fails to significantly reverse muscle atrophy (36, 55, 56). In contrast, MG53 directly induces muscle atrophy via the TLR4/PI3K/AKT/FOXO3a pathway, as demonstrated in our study. The TLR4 pathway has been implicated in muscle wasting induced by lipopolysaccharide (LPS) and other inflammatory stimuli (57). Furthermore, the PI3K/AKT pathway is a master regulator of muscle protein synthesis, and its suppression leads to FOXO3a-mediated activation of the ubiquitin-proteasome system (58, 59). To our knowledge, MG53 is the first secreted protein shown to simultaneously exert antitumor activity and directly induce muscle atrophy through a defined receptor-mediated pathway. This unique feature suggests that MG53 may serve as a critical link between tumor growth and host muscle wasting, a concept that warrants further investigation. This dual role presents a critical translational dilemma: while MG53 suppresses tumor growth, it simultaneously promotes muscle wasting. If MG53 is therapeutically targeted to alleviate cachexia, its antitumor benefits may be compromised. Conversely, approaches that enhance MG53 activity to combat tumors could exacerbate cachexia. Therefore, any therapeutic strategy targeting MG53 must carefully balance these opposing effects. This challenge is not unique to MG53—similar dualities have been observed for other cytokines such as IL-6, which exerts both pro-tumor and cachectic effects (60–62). However, the direct receptor-mediated mechanism of MG53 may offer unique opportunities for selective intervention.

A key finding of our study is that serum MG53 levels are significantly elevated in patients with gastrointestinal cancer cachexia, suggesting its potential as a diagnostic biomarker. Currently, several molecules have been proposed as cachexia biomarkers, including growth differentiation factor 15 (GDF-15), activin A, and IL-6 (63–65). GDF-15 has been shown to induce anorexia and weight loss in preclinical models, and its serum levels correlate with cachexia severity in patients (64). Activin A, a member of the TGF-β superfamily, promotes muscle atrophy through activin type II receptors and has been detected at elevated levels in cachectic patients (66). While these markers are promising, their specificity for cachexia versus other chronic diseases remains a concern. Our data suggest that MG53 may offer a distinct advantage: its upregulation appears to be prominently associated with cachectic patients, particularly those with gastrointestinal cancer, although it is also elevated in other diseases such as diabetes and myocardial infarction. Future studies investigating the combination of MG53 with other cachexia-specific markers are warranted to improve diagnostic accuracy. Moreover, the mechanistic link between MG53 and muscle atrophy via the TLR4 pathway provides a direct functional basis for its biomarker role. Further studies with larger cohorts, including patients with other cancer types such as lung cancer cachexia, are needed to validate the sensitivity and specificity of MG53 as a diagnostic biomarker. Multicenter prospective studies will also be essential to establish MG53 as a clinically useful tool for early detection and monitoring of cancer cachexia.

Our results demonstrate that MG53 induces muscle atrophy through the TLR4/PI3K/AKT/FOXO3a signaling pathway. This finding adds to the growing body of evidence implicating TLR4 in muscle atrophy. TLR4 activation by LPS has been shown to induce muscle atrophy via MyD88-dependent and independent pathways, leading to increased expression of muscle-specific E3 ubiquitin ligases such as MuRF1 and MAFbx (57, 67). In the context of cancer cachexia, TLR4 signaling has been reported to mediate muscle wasting in response to tumor-derived factors (68). The PI3K/AKT pathway is a central regulator of muscle mass: AKT phosphorylation promotes protein synthesis via mTORC1 and inhibits FOXO3a-mediated transcription of atrophy-related genes (58, 69). FOXO3a, in turn, directly upregulates the expression of MuRF1 and MAFbx, driving proteasomal degradation of muscle proteins (58, 70). Notably, the TLR4 pathway can also crosstalk with other cachexia-associated signaling cascades, including p38 MAPK, NF-κB, and STAT3 (57). For example, NF-κB activation has been shown to promote muscle atrophy by upregulating MuRF1 expression (71), while STAT3 signaling is involved in the regulation of muscle protein turnover (72). Whether MG53-mediated TLR4 activation also engages these downstream pathways remains to be elucidated. Future studies should investigate the potential interplay between TLR4 and other inflammatory signaling networks in the context of MG53-induced muscle atrophy, which may reveal additional therapeutic targets.

Several limitations of our study should be acknowledged, along with potential strategies to address them in future research. First, we used CT26 cell supernatant as a cachexia-conditioned medium. While this approach allowed us to identify MG53 as a key cachectic factor, tumor-derived exosomes have been increasingly recognized as important mediators of intercellular communication in cancer cachexia. Exosomes can carry a variety of bioactive molecules, including proteins, mRNAs, and miRNAs, and have been shown to directly induce muscle atrophy (73). Notably, exosomal Hsp70 and Hsp90 have been reported to activate TLR4 signaling in muscle cells (74, 75). Therefore, future studies should investigate whether MG53 is enriched in CT26-derived exosomes and whether exosomal MG53 exerts a more potent or stable effect on muscle atrophy compared with soluble MG53. Second, our findings are based on a single CT26 tumor-bearing mouse model. The cachexia phenotype varies across different tumor models due to differences in tumor growth rate, secretory factor profiles, and host immune responses (76, 77). For instance, although some studies have reported that MC38 can induce tumor cachexia (78). We found that MC38 cells did not induce cachexia under our experimental conditions, which may be related to the relatively short observation period (20 days) and the age of the mice used in our study”. Future studies should include additional cachexia models, such as patient-derived xenograft models or the C26 model, to validate the generalizability of our conclusions. Third, whether MG53 levels are elevated in the serum of patients with other types of cancer cachexia, such as lung, pancreatic, or colorectal cancer, requires further investigation. Fourth, all animal experiments in this study were conducted using young male mice (6–8 weeks old), whereas the clinical cancer cachexia population includes female, elderly, and mixed-gender patients. This represents a significant limitation, as sex and age are known to influence muscle metabolism, inflammatory responses, and tumor-host interactions (79, 80). For instance, estrogen has been shown to modulate muscle wasting pathways, and aged mice exhibit different responses to cachectic stimuli compared with young mice (79, 81). Therefore, future studies should include female and aged mice to validate the generalizability of our findings. In addition, clinical studies should stratify patients by sex and age to determine whether MG53 levels and its role in cachexia vary across these subgroups. Finally, the dual role of MG53 in suppressing tumor growth while promoting muscle wasting presents a major challenge for clinical translation. To resolve this duality, several concrete therapeutic strategies can be considered. First, tumor-specific targeting of MG53 could be achieved through local delivery approaches. Our finding that peritumoral injection of rhMG53 did not induce significant body weight loss (**Supplementary Figure 6**) suggests that localized administration may preserve antitumor efficacy while minimizing systemic cachectic effects. Future studies should explore peritumoral or intratumoral delivery of MG53-based therapies. Second, selective inhibition of the downstream cachexia pathway without affecting MG53’s antitumor activity represents a promising approach. Since MG53 induces muscle atrophy through the TLR4/PI3K/AKT/FOXO3a pathway, blocking this specific signaling cascade in muscle, for example, by using muscle-specific FOXO3a inhibitors or TLR4 antagonists, could potentially mitigate cachexia while preserving MG53’s antitumor effects in the tumor microenvironment. Third, combining MG53-targeted therapy with existing anti-cachexia treatments may offer a synergistic benefit. For instance, MG53 neutralizing antibodies could be administered at low doses that partially reduce MG53 activity—enough to alleviate cachexia without completely abolishing its antitumor effect. Alternatively, MG53-based therapy could be combined with appetite stimulants (e.g., ghrelin agonists) or anabolic agents (e.g., selective androgen receptor modulators) to counteract muscle wasting. Fourth, temporal separation of treatment phases could be considered: initial MG53-based antitumor therapy followed by anti-cachexia intervention once tumor burden is reduced. Future preclinical studies should systematically test these strategies in relevant animal models, including female and aged mice, to evaluate the safety and efficacy of each approach. Finding the optimal balance between antitumor efficacy and cachexia mitigation will be essential for successful clinical translation of MG53-based interventions.

## 5 Conclusion

In summary, our study identifies MG53 as a novel cancer cachexia factor driving muscle atrophy in gastrointestinal cancer cachexia and elevated serum MG53 levels correlate with muscle atrophy in patients. Tumor-derived MG53 promotes cancer cachexia-related muscle atrophy by inhibiting the TLR4/PI3K/AKT/FOXO3a signaling pathway. These results highlight MG53 as a potential diagnostic biomarker and therapeutic target for gastrointestinal cancer cachexia, warranting further investigation in clinical settings.

## Data Availability

The datasets presented in this study can be found in online repositories. The names of the repository/repositories and accession number(s) can be found in the article/**Supplementary Material**.
