# Supplementary material for: Tumor-derived MG53 aggravates gastrointestinal cancer cachexia related muscle atrophy by inhibiting the TLR4/PI3K/AKT/FOXO3a signaling pathway
Source: Front Oncol. 2026 Sep 7;16:1919475. doi: 10.3389/fonc.2026.1919475 (PMC13590720; doi:10.3389/fonc.2026.1919475)
Supplement: Supplementary file 1 [file SupplementaryFile1.docx]

Supplementary Material

Supplementary Material for [Tumor-derived MG53 aggravates gastrointestinal cancer cachexia related muscle atrophy by inhibiting the TLR4/PI3K/AKT/FOXO3a signaling pathway]
**Supplementary Figure 1.** WB analysis of muscle tissue (western blot validation of mouse muscle tissue after intraperitoneal injection of rhMG53 protein).

A and B: WB analysis of gastrocnemius muscle from mice bearing CT26-shMG53 subcutaneous tumors; C and D: WB analysis of gastrocnemius muscle from mice bearing CT26-OE-MG53 subcutaneous tumors; E and F: WB analysis of gastrocnemius muscle from mice bearing MC38-OE-MG53 subcutaneous tumors; G and H: WB analysis of gastrocnemius muscle from BALB/c mice intraperitoneally injected with rhMG53; I and J: WB analysis of tibialis anterior muscle from C57BL/6 mice intraperitoneally injected with rhMG53; K and L: WB analysis of gastrocnemius muscle from C57BL/6 mice intraperitoneally injected with rhMG53. Results are expressed as mean ± SD. Differences between groups were analyzed for statistical significance using one-way ANOVA followed by post-hoc multiple comparison tests (Tukey's HSD test) with Prism version 8.0 (GraphPad, CA, USA). For all analyses, * p < 0.05, ** p < 0.01, *** p < 0.001, **** p < 0.0001.

**Supplementary Figure 2.** **HE staining of muscle tissues.**

A: Representative H&E staining images of gastrocnemius muscles from BALB/c mice after intraperitoneal injection of rhMG53 protein. B: Representative H&E staining images of tibialis anterior muscles from C57BL/6 mice after intraperitoneal injection of rhMG53 protein. C: Representative H&E staining images of gastrocnemius muscles from C57BL/6 mice after intraperitoneal injection of rhMG53 protein. D: Representative H&E staining images of gastrocnemius muscles from mice bearing CT26-shMG53 subcutaneous xenografts. E: Representative H&E staining images of gastrocnemius muscles from mice bearing CT26-OE-MG53 subcutaneous xenografts. F: Representative H&E staining images of gastrocnemius muscles from mice bearing MC38-OE-MG53 subcutaneous xenografts. Results are expressed as mean ± SD. Differences between groups were analyzed for statistical significance using one-way ANOVA followed by post-hoc multiple comparison tests (Tukey's HSD test) with Prism version 8.0 (GraphPad, CA, USA). For all analyses, * p < 0.05, ** p < 0.01, *** p < 0.001, **** p < 0.0001.

**Supplementary Figure 3.** **Endogenous MG53 can cause atrophy in C2C12 myotubes.**

A: The knockdown efficiency in C2C12-shMG53 cells was verified by WB and qRT-PCR experiments; B: MyHC immunofluorescence staining of C2C12-shMG53 myotubes. Scale bar, 100 μm; C: The diameter and area of immunofluorescence-stained C2C12-shMG53 myotubes were statistically analyzed using Image J software; D: Protein expression levels of MG53, Myogenin, MyHC, MuRF1, and MAFbx in C2C12-shMG53 myotubes were detected by WB. Relative quantification of muscle atrophy- and synthesis-related proteins was performed using ImageJ software. E: The overexpression efficiency in C2C12-OE-MG53 cells was verified by WB and qRT-PCR experiments; F: MyHC immunofluorescence staining of C2C12-OE-MG53 myotubes. Scale bar, 100 μm; G: The diameter and area of immunofluorescence-stained C2C12-OE-MG53 myotubes were statistically analyzed using ImageJ software. H: Protein expression levels of MG53, Myogenin, MyHC, MuRF1, and MAFbx in C2C12-OE-MG53 myotubes were detected by WB. Relative quantification of muscle atrophy- and synthesis-related proteins was performed using ImageJ software. Results are expressed as mean ± SD. One-way ANOVA followed by post-hoc multiple comparisons test (Tukey’s HSD test) using Prism version 8.0 (GraphPad, CA, USA) was used to analyze the statistical significance of differences between groups. For all analyses, * p < 0.05, ** p < 0.01, *** p < 0.001, **** p < 0.0001.

**Supplementary Figure 4.** **WB analysis of muscle tissue (western blot validation of muscle tissue from mice with subcutaneous tumors and MG53 knockdown or overexpression).**

A and B: WB analysis of gastrocnemius muscle from BALB/c mice intraperitoneally injected with rhMG53; C and D: WB analysis of tibialis anterior muscle from C57BL/6 mice intraperitoneally injected with rhMG53; E and F: WB analysis of gastrocnemius muscle from C57BL/6 mice intraperitoneally injected with rhMG53. Results are expressed as mean ± SD. Differences between groups were analyzed for statistical significance using one-way ANOVA followed by post-hoc multiple comparison tests (Tukey's HSD test) with Prism version 8.0 (GraphPad, CA, USA). For all analyses, * p < 0.05, ** p < 0.01, *** p < 0.001, **** p < 0.0001.

**Supplementary Figure 5.** **Antitumor effects of MG53 in vitro.**

A: Representative bright-field images of CT26 cells treated with rhMG53 protein at different time points; B: Representative bright-field images of MC38 cells treated with rhMG53 protein at different time points; C and D: Colony formation images of MG53-knockdown CT26 cells, with quantification of colony number and area; E and F: Colony formation images of MG53-overexpressing CT26 cells, with quantification of colony number and area; G and H: Colony formation images of MG53-overexpressing MC38 cells, with quantification of colony number and area; I: Statistical analysis of CCK8 assay for MG53-knockdown CT26 cells; J: Statistical analysis of CCK8 assay for MG53-overexpressing CT26 cells; K: Statistical analysis of CCK8 assay for MG53-overexpressing MC38 cells; L: Statistical analysis of CCK8 assay evaluating the cytotoxic effect of rhMG53 protein on CT26 cells; M: Statistical analysis of CCK8 assay evaluating the cytotoxic effect of rhMG53 protein on MC38 cells. Results are expressed as mean ± SD. Differences between groups were analyzed for statistical significance using one-way ANOVA followed by post-hoc multiple comparison tests (Tukey's HSD test) with Prism version 8.0 (GraphPad, CA, USA). For all analyses, * p < 0.05, ** p < 0.01, *** p < 0.001, **** p < 0.0001.

**Supplementary Figure 6.** **Antitumor effects of MG53 in vivo.**

A: Tumors in BALB/c mice with subcutaneous CT26 cell xenografts following peritumoral injection of rhMG53 protein. Control group received an equal volume of PBS. Representative tumor images at the end of the experiment (n=6). B: Subcutaneous tumor weight (n=10). C: Subcutaneous tumor growth curve (n=10). D: Body weight change curve of tumor-bearing mice (n=10). E: Tumor-free body weight of tumor-bearing mice (n=10). F: Food consumption of tumor-bearing mice (n=10). G: Weight of tibialis anterior and gastrocnemius muscles from tumor-bearing mice (n=10). H: Tumors in C57BL/6 mice with subcutaneous MC38 cell xenografts following peritumoral injection of rhMG53 protein. Control group received an equal volume of PBS. Representative tumor images at the end of the experiment (n=6). B: Subcutaneous tumor weight (n=8). (Note: The repeated use of letters B-G is incorrect for figure labeling.) C: Subcutaneous tumor growth curve (n=8). D: Body weight change curve of tumor-bearing mice (n=8). E: Tumor-free body weight of tumor-bearing mice (n=8). F: Food consumption of tumor-bearing mice (n=8). G: Weight of tibialis anterior and gastrocnemius muscles from tumor-bearing mice (n=8). Results are expressed as mean ± SD. Differences between groups were analyzed for statistical significance using one-way ANOVA followed by post-hoc multiple comparison tests (Tukey's HSD test) with Prism version 8.0 (GraphPad, CA, USA). For all analyses, * p < 0.05, ** p < 0.01, *** p < 0.001, **** p < 0.0001.
